# Supplementary material for: Targeting advanced prostate cancer with STEAP1 chimeric antigen receptor T cell and tumor-localized IL-12 immunotherapy
Source: Nat Commun. 2023 Apr 11;14:2041. doi: 10.1038/s41467-023-37874-2 (PMC10090190; doi:10.1038/s41467-023-37874-2)
Supplement: Supplementary file 1 — Supplementary Information [file 41467_2023_37874_MOESM1_ESM.pdf]

## Supplementary Information

# Targeting advanced prostate cancer with STEAP1 chimeric antigen receptor T cell and tumor-localized IL-12 immunotherapy

Vipul Bhatia[1,*], Nikhil V. Kamat[2,*], Tiffany E. Pariva[1,*], Li-Ting Wu[1], Annabelle Tsao[1], Koichi Sasaki[3], Huiyun Sun[1], Gerardo Javier[1], Sam Nutt[1], Ilsa Coleman[1], Lauren Hitchcock[1], Ailin Zhang[1], Dmytro Rudoy[1], Roman Gulati[4], Radhika A. Patel[1], Martine P. Roudier[5], Lawrence D. True[6], Shivani Srivastava[1], Michael C. Haffner[1,6,7], Peter S. Nelson[1,2,4,5,6,7], Saul J. Priceman[8,9], Jun Ishihara[3,#], John K. Lee[1,2,6,7,#]

[1] Human Biology Division, Fred Hutchinson Cancer Center. 1100 Fairview Ave N, Seattle, WA 98109, USA.
[2] Division of Medical Oncology, University of Washington. 1959 NE Pacific Street, Seattle, WA, 98195, USA.
[3] Department of Bioengineering, Imperial College London. 86 Wood Lane, London W12 0BZ, UK.
[4] Public Health Sciences Division, Fred Hutchinson Cancer Center. 100 Fairview Ave N, Seattle, WA 98109, USA.
[5] Department of Urology, University of Washington. 1959 NE Pacific Street, Seattle, WA, 98195, USA.
[6] Department of Pathology and Laboratory Medicine, University of Washington. 1959 NE Pacific Street, Seattle, WA 98195, USA.
[7] Clinical Research Division. Fred Hutchinson Cancer Center. 1100 Fairview Ave N, Seattle, WA 98109, USA.
[8] Department of Hematology and Hematopoietic Cell Transplantation, City of Hope. 1500 East Duarte Road, Duarte, CA 91010, USA.
[9] Department of Immuno-Oncology, Beckman Research Institute of City of Hope. 1500 East Duarte Road, Duarte, CA 91010, USA.

[*] These authors contributed equally to this work.

[#] Corresponding authors:
Jun Ishihara j.ishihara@imperial.ac.uk
John K. Lee jklee5@fredhutch.org

## Supplementary Information includes:

Supplementary Figures S1 to S19

## Other Supplementary Materials for this manuscript include:

Source Data file

## Supplementary Figure 1

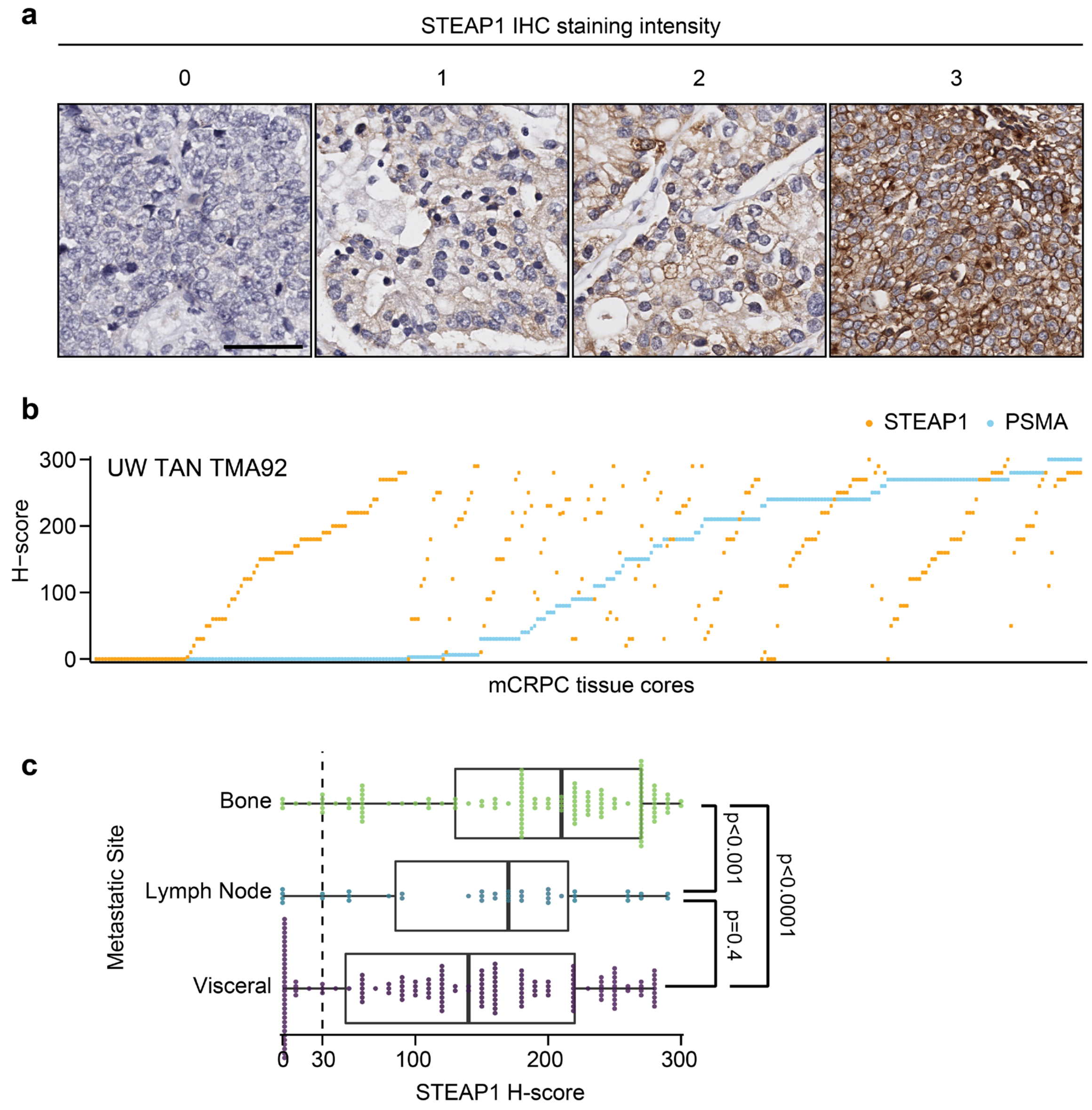


**d**

| Characteristic | PSMA, n = 44 | STEAP1, n = 44 | p-value |
|---|---|---|---|
| H-score, median (IQR) | 108 (9, 223) | 158 (105, 209) | 0.036[a] |
| H-score >0, n (%) | 37 (84%) | 44 (100%) | <0.001[b] |
| H-score ≥30, n (%) | 30 (68%) | 42 (95%) | <0.001[b] |

[a]Wilcoxon signed rank test with continuity correction
[b]McNemar's Chi-squared test

**Supplementary Figure 1. Characteristics of STEAP1 expression in lethal mCRPC tissues.** (**a**) Photomicrographs of select mCRPC tissue cores after STEAP1 IHC staining to highlight the plasma membrane staining consistent with staining intensity scores of 0, 1, 2, and 3. Scale bars = 50 μm. (n=332 mCRPC cores were stained for STEAP1) (**b**) Plot showing paired H-scores of STEAP1 (blue) and PSMA (orange) immunohistochemical (IHC) staining of all cores from each mCRPC tissue. (**c**) Boxplots of STEAP1 H-scores of mCRPC tissue cores based on their metastatic site (n=332 tissue cores) show the median (thick vertical line), interquartile range spanning the 25th to the 75th

percentiles (thin rectangle), and the smallest and largest values that lie within 1.5 times the interquartile range of the 25th and 75th percentiles (horizontal “whiskers”). Superimposed dots show STEAP1 H-scores for individual tissue cores. Two-sided p-values were obtained from a linear mixed model with untransformed H-scores with random effect for patients. No adjustments were made for multiple comparisons. P-values for bone vs. visceral and bone vs. lymph nodes are <0.0001. (**d**) Patient-level analysis of median H-scores (averaged within patients) using a Wilcoxon signed rank test and for the frequency of STEAP1 or PSMA IHC staining above the specificed H-score thresholds (p<0.001 for H-score ≤30 and >0) using McNemar’s Chi-squared test. No adjustments were made for multiple comparisons. All p-values were two-sided. Source data are provided in the Source Data file.

## Supplementary Figure 2

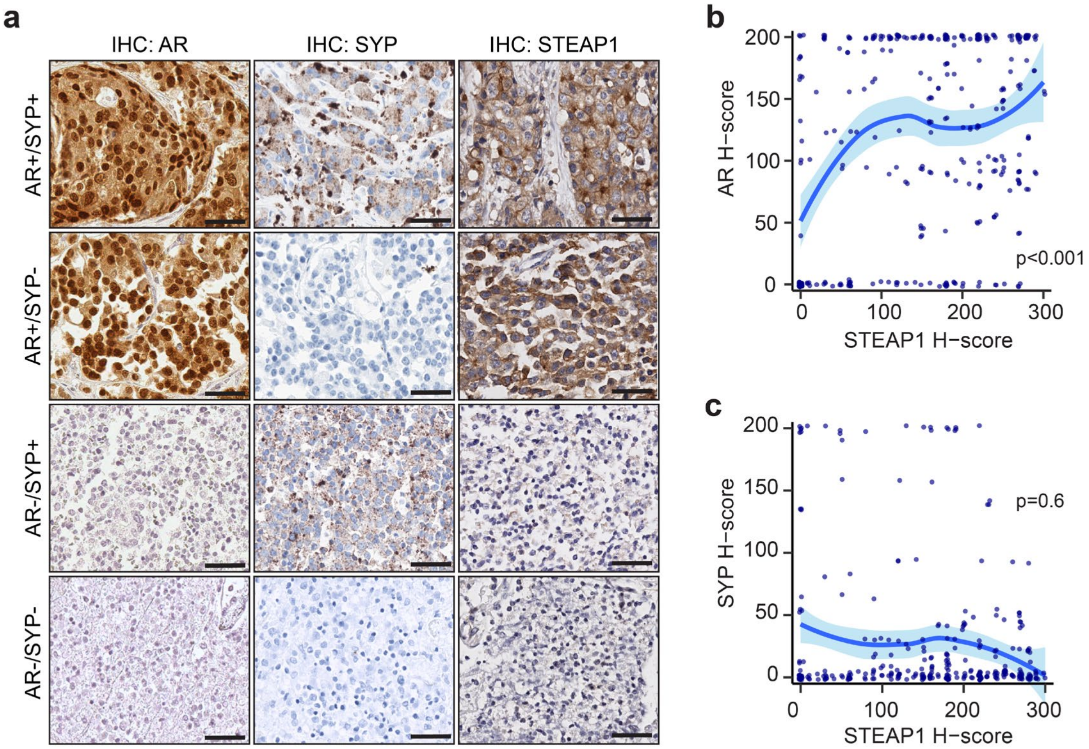


**Supplementary Figure 2. STEAP1 expression positively correlates with androgen receptor expression in mCRPC.** (**a**) Photomicrographs of androgen receptor (AR), synaptophysin (SYP), and STEAP1 IHC staining in representative sections from AR+/SYP+, AR+/SYP-, AR-/SYP+ and AR-/SYP- mCRPC tissues (n=332 mCRPC cores were stained for AR, SYP, STEAP1 and PSMA). Scatter plots of STEAP1 H-score and (**b**) AR H-score ($p<0.001$) or (**c**) SYP H-score ($p=0.6$) for each mCRPC tissue core (points), jittered to reduce overplotting, and a fitted local polynomial regression (smooth curve) with pointwise 95% confidence limits (semitransparent ribbon). Two-sided p-values in panels (**b**) and (**c**) were obtained from separate fitted linear mixed models with random effect for patients. applied to log-transformed H-scores after adding 1 to each H-score (to avoid taking the log of zero). No adjustments were made for multiple comparisons. Source data are provided in the Source Data file.

## Supplementary Figure 3

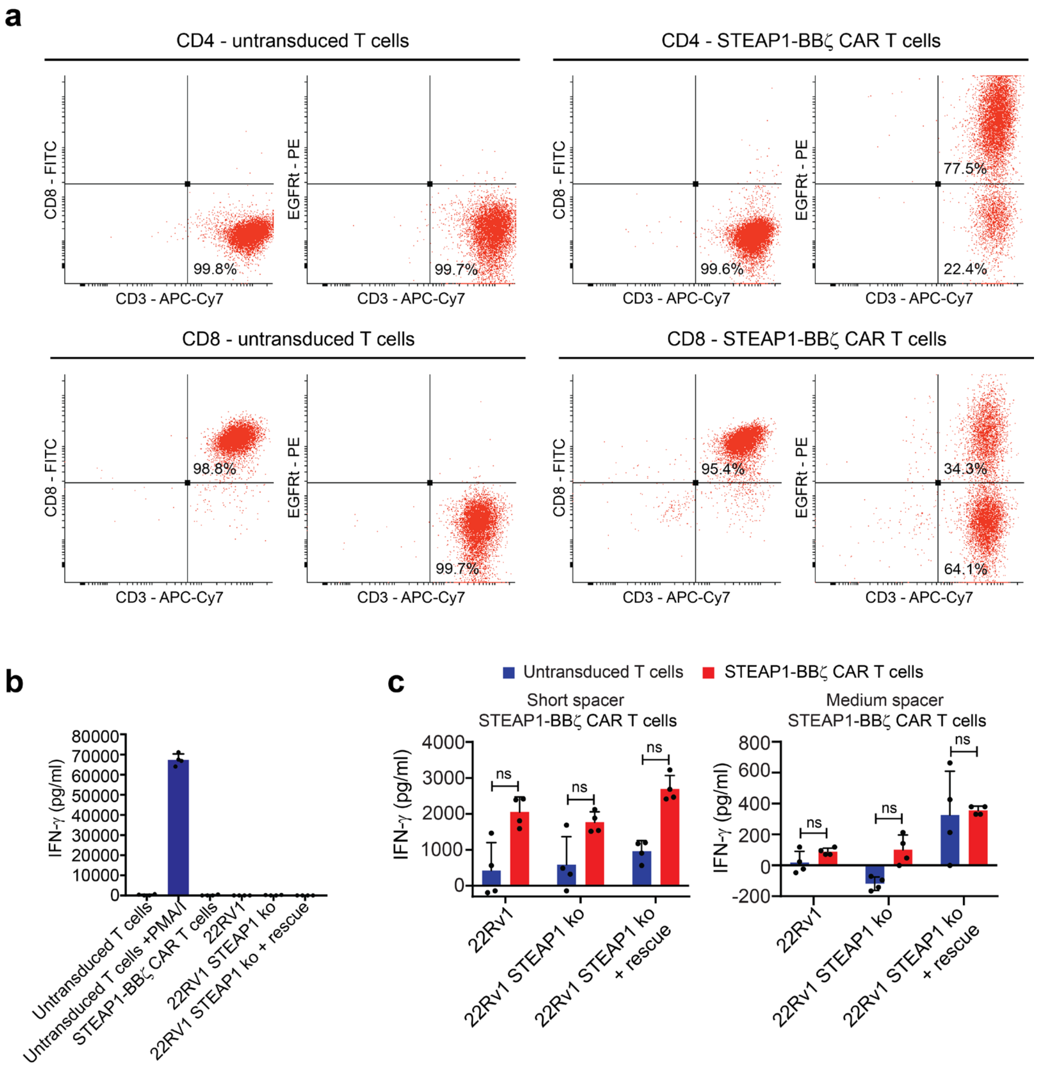


**Supplementary Figure 3. Validation of the antigen-specific activation and target cell cytolysis of STEAP1-BBζ CAR T cells.** (**a**) Representative flow cytometry plots showing immunophenotyping of CD4 and CD8 T cell products at day 9 of expansion from untransduced control and lentiviral STEAP1 CAR transduction conditions. IFN-γ quantification by ELISA from (**b**) control culture conditions including untransduced T cells with Phorbol-Myristate-Acetate and Ionomycin (PMA/I) and only target cells conditions. (**c**) IFN-γ quantification by ELISA from co-cultures of either untransduced T cells or short (left) and medium (right) spacer length STEAP1-BBζ CAR T cells with parental 22Rv1, 22Rv1 STEAP1 knockout (ko) cells, and 22Rv1 STEAP1 ko with STEAP1 rescue cells at a 1:1 ratio at 24 hours. n=4 biological replicates per condition. Bars represent SEM. P-values were estimated using unpaired two-tailed Student's t test with Welch's correction. Source data are provided in the Source Data file.

## Supplementary Figure 4

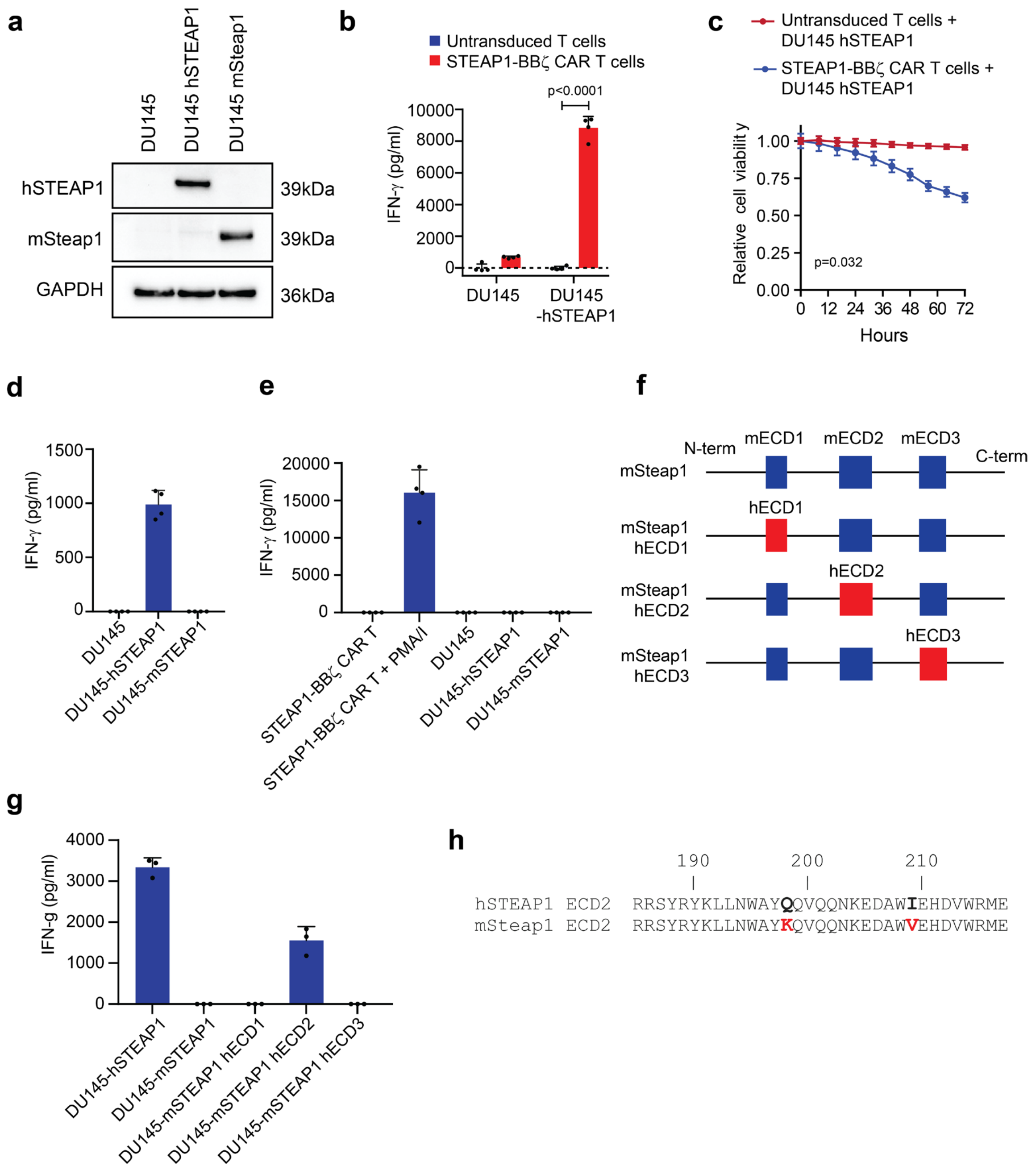


**Supplementary Figure 4. Determination of the STEAP1 ectodomain specificity of STEAP1-BBζ CAR T cells using mouse/human Steap1 chimeras.** (**a**) Immunoblot analysis confirming the lack of human STEAP1 (hSTEAP1) and mouse Steap1 (mSteap1) expression in the DU145 cell line and their respective expression in the lentivirally engineered DU145 hSTEAP1 and DU145 mSteap1 lines. GAPDH is used as a protein loading control. n=3 biologically independent experiments were performed. (**b**) IFN-γ quantification by ELISA from co-cultures of STEAP1-BBζ CAR T cells with parental DU145 and DU145 hSTEAP1 cells. n=4 replicates per condition. Bars represent SD. (**c**) Relative cell viability of DU145 and DU145 hSTEAP1 target cells over time measured by fluorescence live cell imaging upon co-culture with STEAP1-BBζ CAR T cells or untransduced T cells at a 1:1 ratio. n=4 biological replicates per condition. Bars represent mean with SEM. P-values were estimated using unpaired two-

tailed Student's t test with Welch's correction. (**d**) IFN-γ quantification by ELISA from co-cultures of STEAP1-BBζ CAR T cells with the DU145, DU145 hSTEAP1, and DU145 mSteap1 cell lines at a 1:1: ratio or (**e**) control culture conditions at 24 hours. n = 4 biological replicates per condition. For panels (**d-e**) bars represent mean with SEM. (**f**) Schematic of the mSteap1 protein with extracellular domains (mECDs, blue) highlighted and the mouse/human Steap1 chimeric proteins each with individual replacement of a mECD with the counterpart hSTEAP1 extracellular domain (hECD, red). (**g**) IFN-γ quantification by ELISA from co-cultures of STEAP1-BBζ CAR T cells with each of the DU145 lines engineered to express hSTEAP1, mSteap1, or mouse/human Steap1 chimeric proteins at a 1:1 ratio at 24 hours. n=4 biological replicates per condition. Bars represent mean with SD. (**h**) Alignment of the amino acid sequences of the hSTEAP1 ECD2 and mSteap1 ECD2 showing non-conserved residues at positions 198 and 209. Source data are provided in the Source Data file.

## Supplementary Figure 5

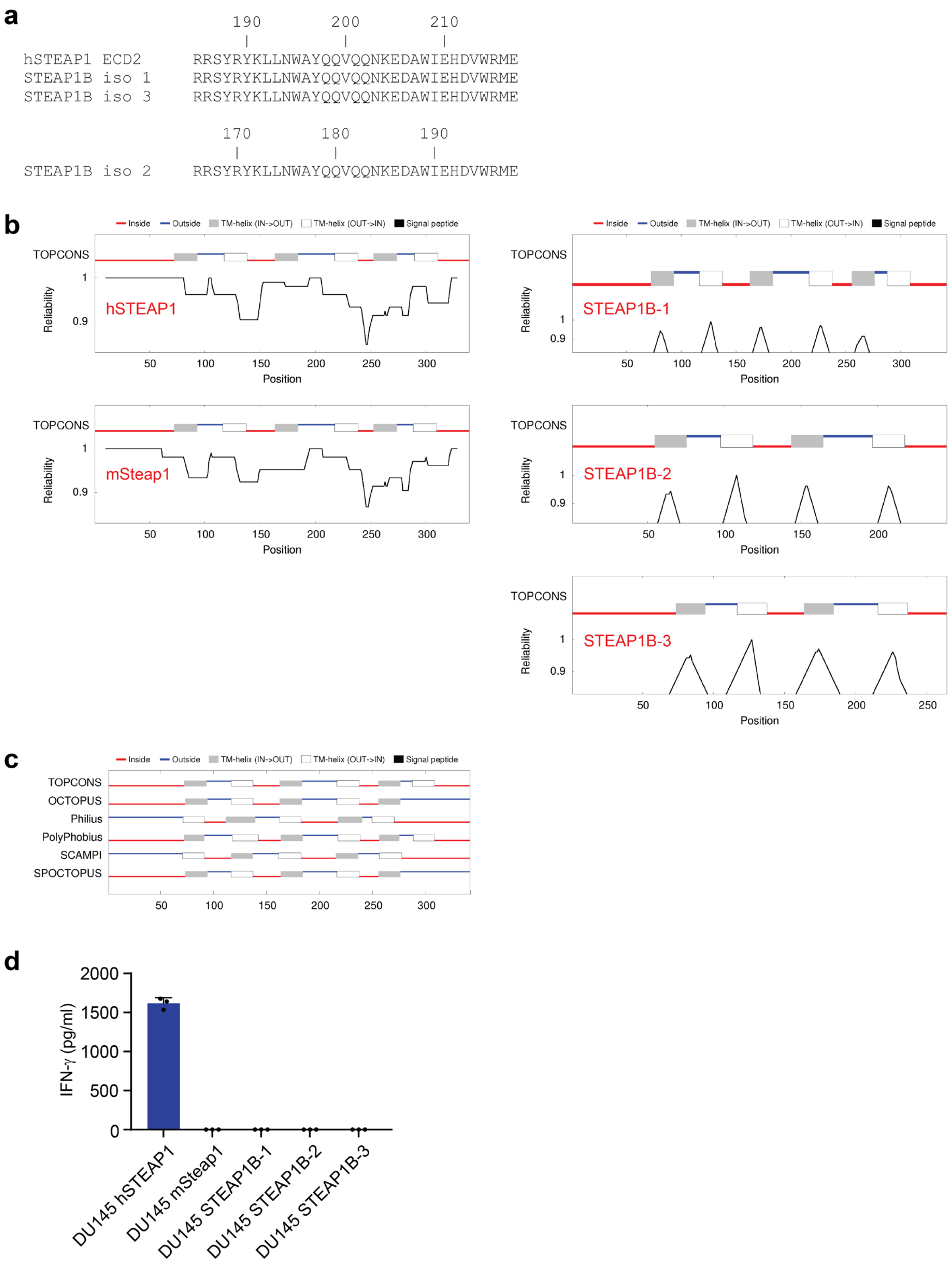


**Supplementary Figure 5. Evaluation of the reactivity of STEAP1-BBζ CAR T cells to STEAP1B isoforms.** (**a**) Alignment of the amino acid sequence of the hSTEAP1 ECD2 to human STEAP1B isoforms 1, 2, and 3 showing complete sequence conservation. (**b**) Plots of TOPCONS consensus predictions of membrane protein topology and reliability scores for hSTEAP1, mSteap1, and the three human STEAP1B isoforms. (**c**) Plots of membrane protein topology predictions for human STEAP1B

isoform 1 showing disagreement between different algorithms. (**d**) IFN-γ quantification by ELISA from co-cultures of STEAP1-BBζ CAR T cells with each of the DU145 lines engineered to express hSTEAP1, mSteap1, or human STEAP1B isoforms at a 1:1 ratio at 24 hours. n=4 biological replicates per condition. Bars represent mean with SD. Source data are provided in the Source Data file.

## Supplementary Figure 6

**a**

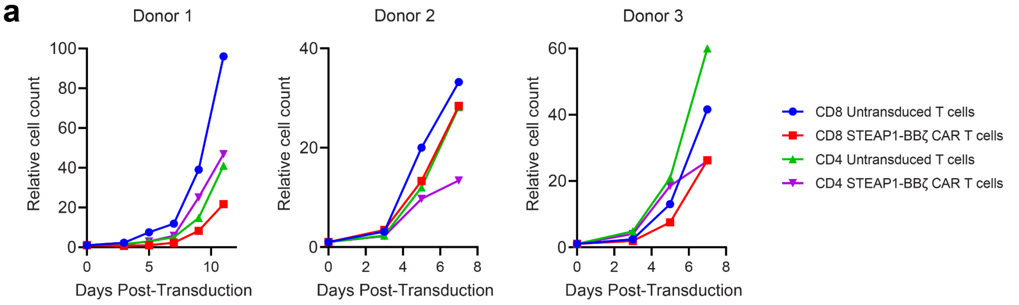


**b**

| | CD4 EGFRt+ | CD8 EGFRt+ |
|---|---|---|
| Donor 1 | 60.1% | 26.2% |
| Donor 2 | 74.9% | 24.3% |
| Donor 3 | 69.3% | 54.2% |

**c**

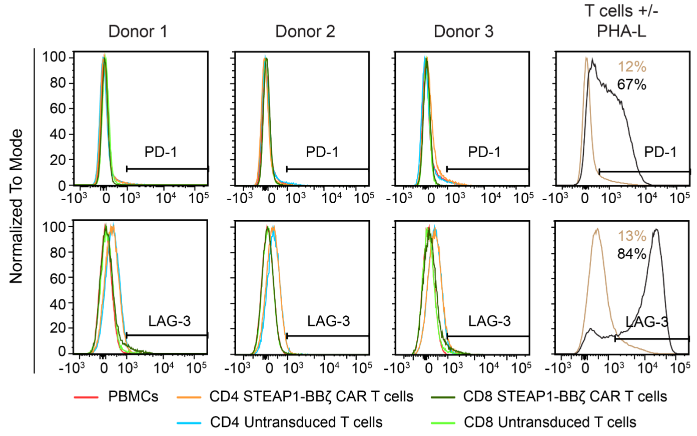


**d**

**e**

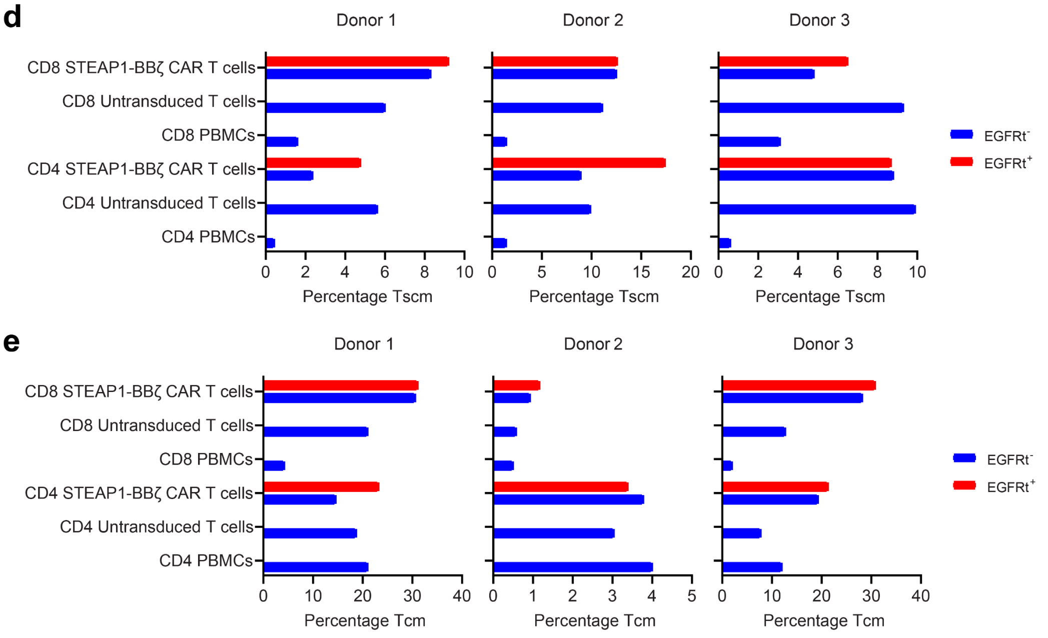


**Supplementary Figure 6. Characterization of the expansion, transduction efficiency, and differentiation states of STEAP1-BBζ CAR T cell products.** (**a**) Plots showing the relative expansion of untransduced and STEAP1-BBζ CAR T cell subsets derived from three independent donors over time in culture. (**b**) Table showing the percentage of T cell subsets transduced with STEAP1-BBζ CAR lentivirus that express EGFRt as measured by flow cytometry seven days after transduction. (**c**) Flow

cytometry histogram plots showing the expression of the T cell exhaustion markers PD-1 and LAG-3 in PBMCs as well as untransduced and STEAP1-BBζ CAR T cell subsets. T cells treated with PHA-L are used as a positive control for induction of PD-1 and LAG-3 expression. Bar graphs showing the percentages of (**d**) stem cell memory T cells (Tscm: $CD62L^{+}$ $CD45RA^{+}$ $CD95^{+}$ $CXCR3^{+}$) and (**e**) central memory T cells (Tcm: $CD62L^{+}$ $CD45RA^{-}$) in T cell subsets from PBMCs as well as untransduced and STEAP1-BBζ CAR T cell products. Source data are provided in the Source Data file.

## Supplementary Figure 7

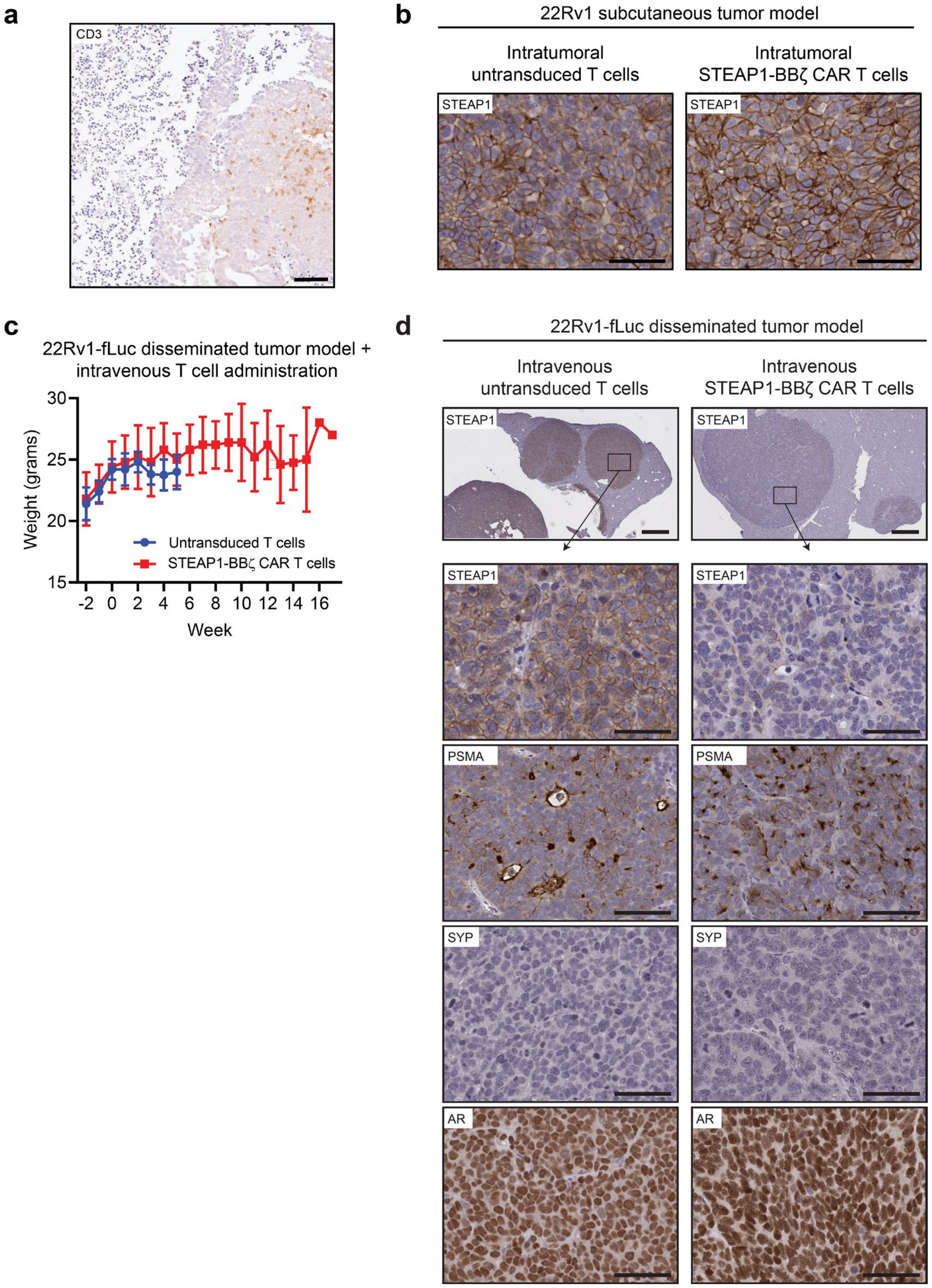


**Supplementary Figure 7. Antitumor activity of STEAP1-BBζ CAR T cell therapy in human 22Rv1 prostate cancer cell line xenograft models.** (**a**) Photomicrograph of CD3 IHC staining of a 22Rv1 subcutaneous tumor 25 days after intratumoral treatment with STEAP1-BBζ CAR T cells. Scale bars = 100 µm. (**b**) Representative photomicrographs of STEAP1 IHC staining of 22Rv1-fLuc subcutaneous tumors after intratumoral treatment with (left) untransduced T cells or (right) STEAP1-BBζ CAR T cells.

Scale bars = 50 μm. For panel (**a-b**) immunostaining was performed on n=4 biologically independent specimens. (**c**) Plot of average weights of NSG mice engrafted wih 22Rv1-fLuc metastatic tumors over time after treatment with either untransduced T cells or STEAP1-BBζ CAR T cells. n=5 mice per group. Bars represent mean with SD. (**d**) Representative low magnification (top) photomicrographs of STEAP1 IHC staining of 22Rv1-fLuc metastatic liver tumors after intravenous treatment with (left) untransduced T cells or (right) STEAP1-BBζ CAR T cells. Scale bars = 1 mm. Higher magnification photomicrographs of STEAP1, PSMA, SYP and AR IHC staining of regions shown below. Scale bars = 50 μm. Source data are provided in the Source Data file.

## Supplementary Figure 8

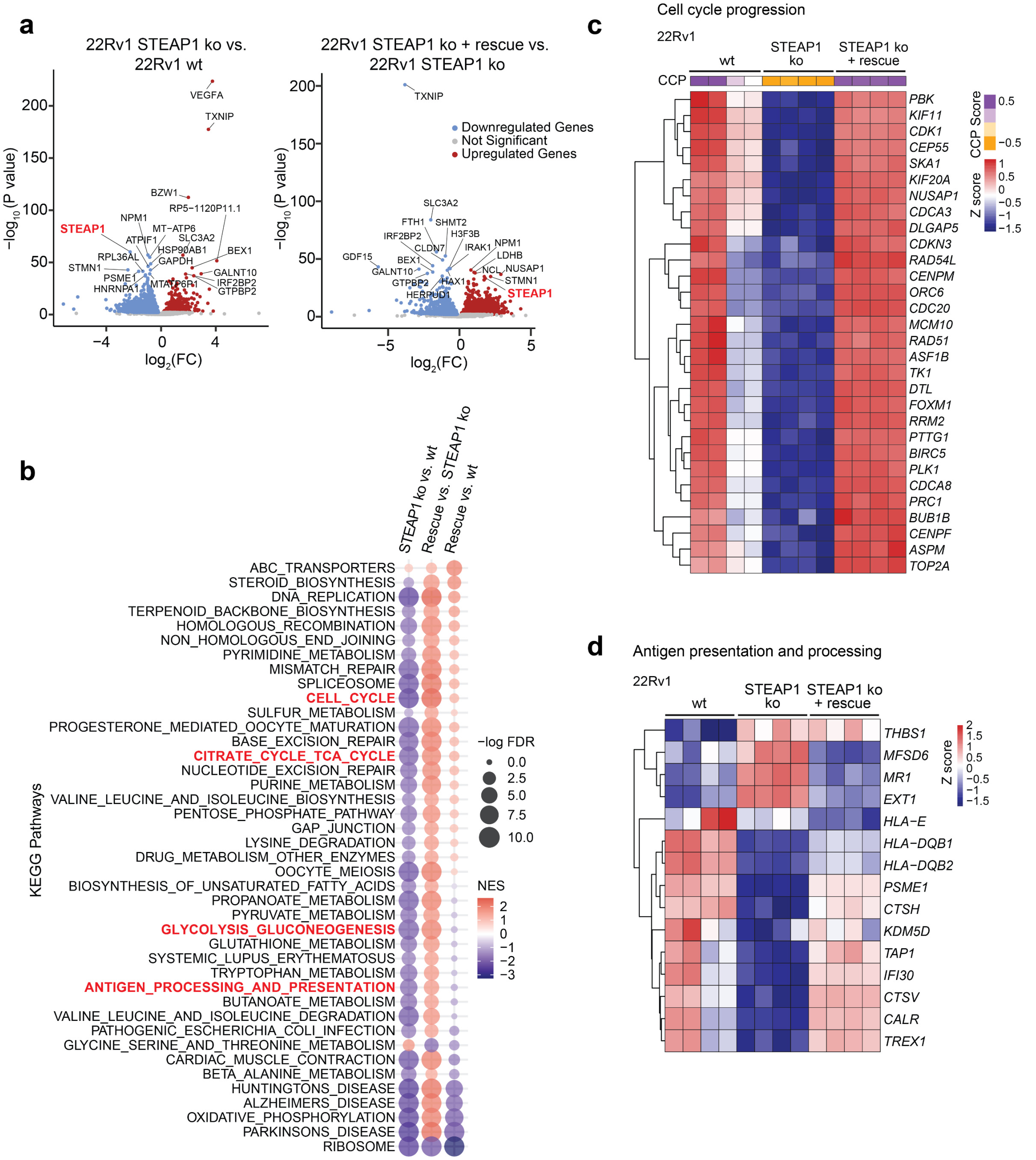


**Supplementary Figure 8. Transcriptome and pathway analysis of isogenic 22Rv1 cell lines with STEAP1 knockout and addback.** (**a**) Volcano plots depicting differentially expressed genes in 22Rv1 STEAP1 knockout (ko) cells compared to 22Rv1 wildtype (wt) cells (left) and in 22Rv1 STEAP1 ko cells with addback of STEAP1 (+ rescue) compared to 22Rv1 STEAP1 ko cells (right). Red and blue dots represent upregulated and downregulated differentially expressed transcripts (DETs), respectively, with adjusted p<0.05 and log2 (fold change) >0.6. Top 20 deregulated genes are marked. Wald-test was used for calculate the p-values with Benjamin-Hochberg correction. (**b**) Bubble plot showing KEGG pathways that are positively and negatively enriched in conditions mentioned in (**a**). The size of the bubbles represents -log FDR indicating the significance of enriched pathways while the color scheme represents the negative or positive normalized enrichment score (NES) predicted by GSEA. (**c**)

Heatmap representing the 31 gene signature used to evaluate the cell cycle progression (CCP) score in 22Rv1 wt, 22Rv1 STEAP1 ko, and 22Rv1 STEAP1 ko + rescue cells. The color schemes represents the Z score and the CCP score for each sample. (**d**) Heatmap showing differentially expressed genes (FDR<0.05) associated with antigen presentation and processing. For panels (**a-d**) data represent n=4 independent biological samples per conditions which were processed for RNA-seq.

## Supplementary Figure 9

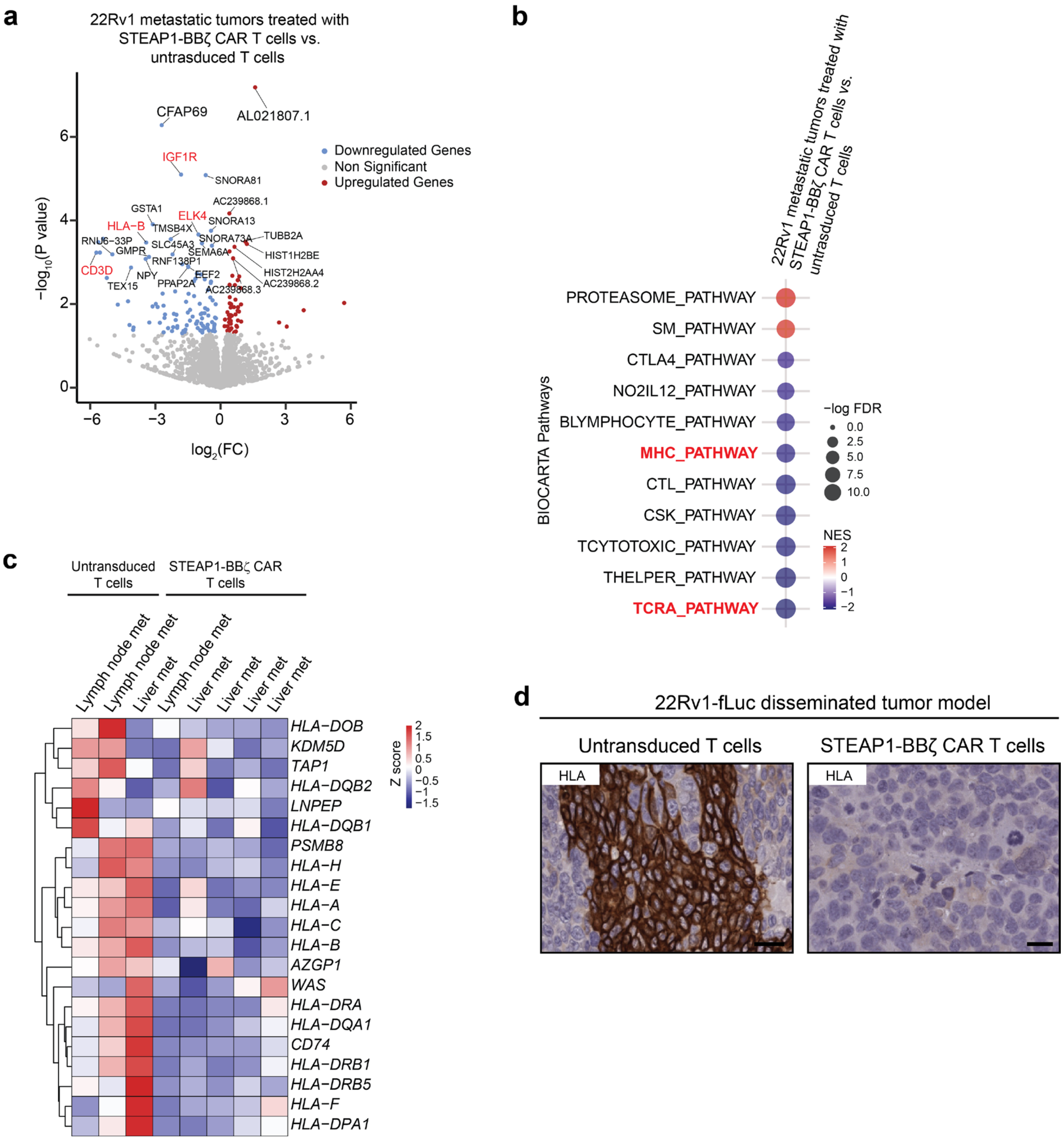


**Supplementary Figure 9. Antigen presentation and processing is negatively enriched in 22Rv1 metastatic tumors with STEAP1 antigen loss after treatment with STEAP1-BBζ CAR T cell therapy.** (**a**) Volcano plots depicting differentially expressed genes in 22Rv1 metastatic tumors from mice treated with STEAP1-BBζ CAR T cells compared to those treated with untransduced T cells. Red and blue dots represent upregulated and downregulated differentially expressed transcripts (DETs), respectively, with adjusted p<0.05 and log2 (fold change) >0.6. The top 20 deregulated genes are marked and those associated with antigen processing and presentation are highlighted with gene names in red. Wald-test was used for calculate the p-values with Benjamin-Hochberg correction. (**b**) Bubble plot showing BIOCARTA pathways that are positively and negatively enriched in conditions mentioned in (**a**). The size of the bubbles represents -log FDR indicating the significance of enriched pathways while the color scheme represents the negative or positive NES predicted by GSEA. (**c**) Heatmap representing the genes associated with antigen presentation and processing in 22Rv1 metastatic tumor samples from mice treated with untransduced T cells and STEAP1-BBζ CAR T cells.

(**d**) Representative photomicrographs of IHC staining for HLA-A,B,C in 22Rv1 metastatic liver tumors from mice treated with untransduced T cells (left) or STEAP1-BBζ CAR T cells (right). Scale bars = 20 µm. Immunostaining was performed on n=3 biologically independent specimens. Source data are provided in the Source Data file.

## Supplementary Figure 10

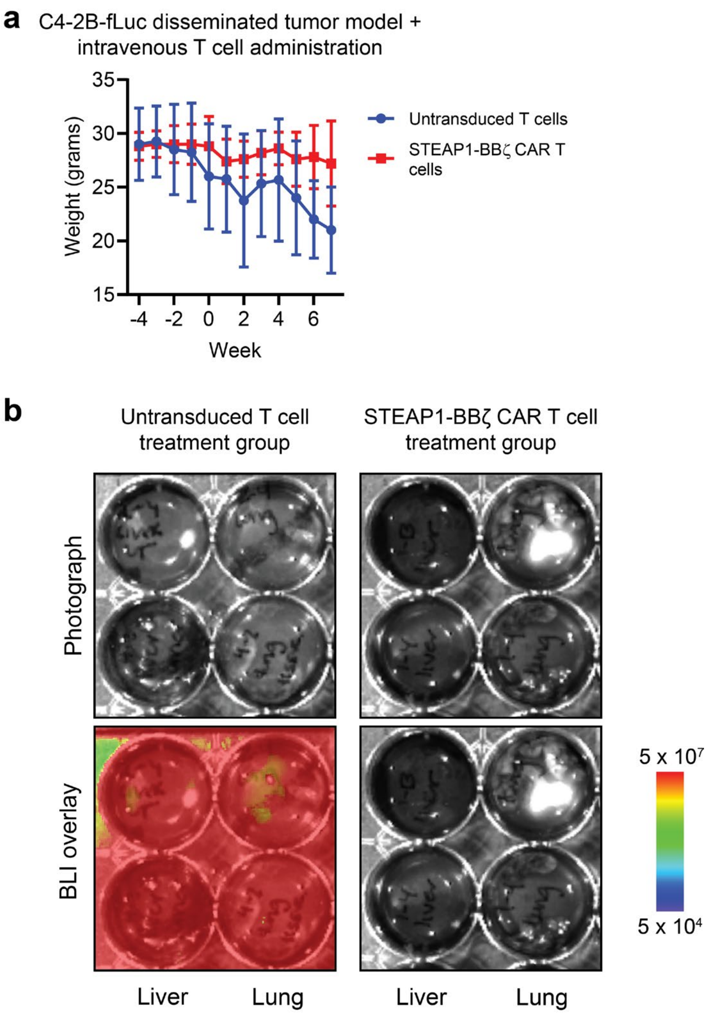


**Supplementary Figure 10. Antitumor activity of STEAP1-BBζ CAR T cell therapy in a disseminated human C4-2B prostate cancer cell line xenograft model.** (**a**) Plot of average weights of NSG mice engrafted wih C4-2B-fLuc metastatic tumors over time after treatment with either untransduced T cells (n=4) or STEAP1-BBζ CAR T cells (n=5). Bars represent SD. (**b**) *Ex vivo* photographic images (top) and BLI overlay (bottom) of individual liver and lung tissues from NSG mice engrafted with C4-2B-fLuc metastatic tumors and treated with either (left) untransduced T cells or (right) STEAP1-BBζ CAR T cells. Panel (**b**) shows n=4 individual tumors in each group. Radiance scale is shown. Source data are provided in the Source Data file.

## Supplementary Figure 11

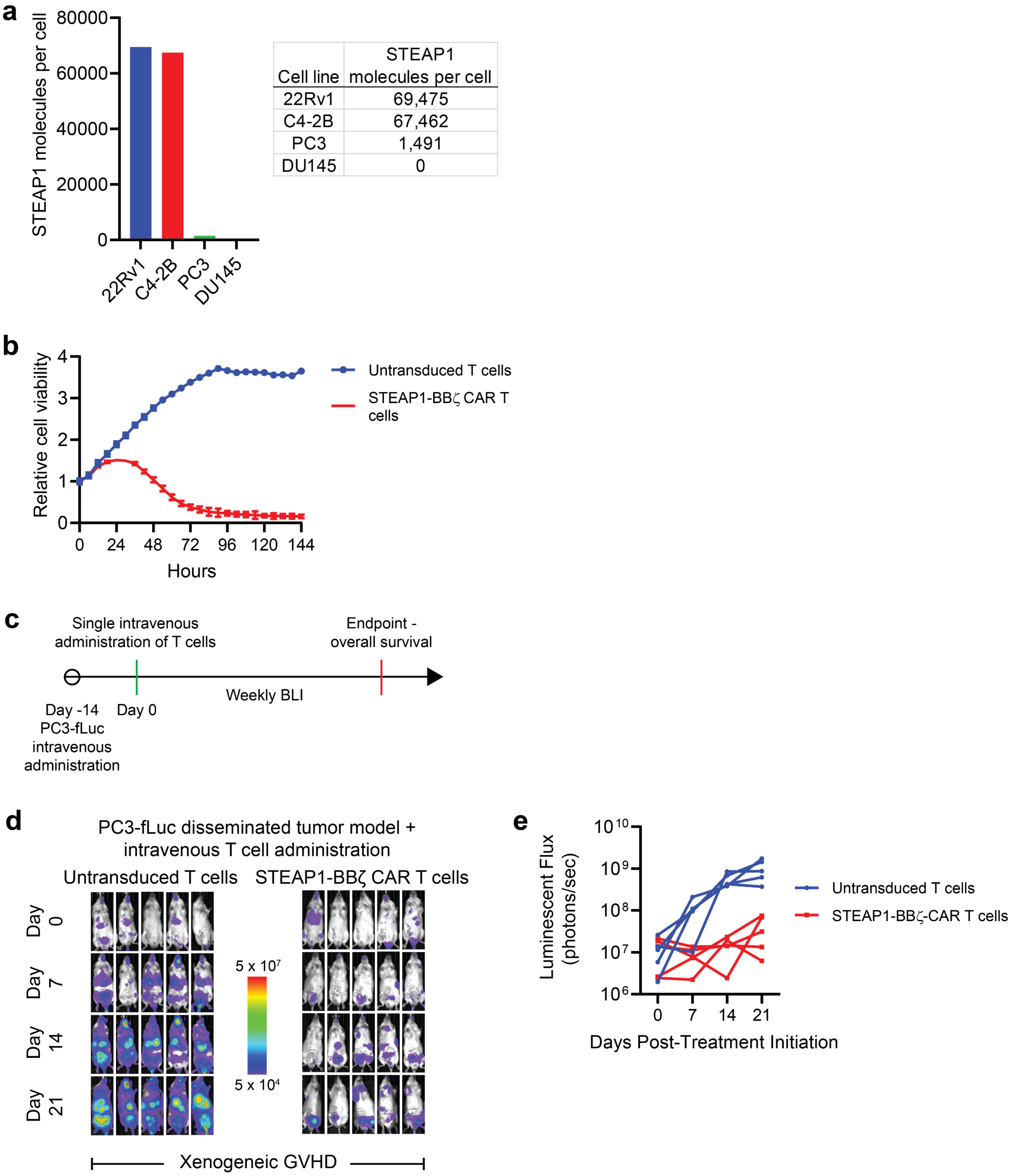


| Cell line | STEAP1 molecules per cell |
|---|---|
| 22Rv1 | 69,475 |
| C4-2B | 67,462 |
| PC3 | 1,491 |
| DU145 | 0 |

**Supplementary Figure 11. Antitumor activity of STEAP1-BBζ CAR T cell therapy in a disseminated human PC3 prostate cancer cell line xenograft model.** (**a**) Plot and table showing absolute quantification of STEAP1 molecules per cell across select human prostate cancer cell lines including PC3 as determined by flow cytometry using external standard beads. (**b**) Relative cell viability of PC3 target cells over time measured by fluorescence live cell imaging upon co-culture with untransduced T cells or STEAP1-BBζ CAR T cells at a 1:1 ratio. n=4 biological replicates per condition and bars represent SEM. (**c**) Schematic of tumor challenge experiments for the PC3 disseminated model. (**d**) Serial live bioluminescence imaging (BLI) of NSG mice engrafted with PC3-fLuc metastases and treated with a single intravenous injection of 5 x $10^6$ untransduced T cells or STEAP1-BBζ CAR T cells at a normal CD4/CD8 ratio on day 0. Mice were euthanized due to the onset of severe xenogeneic

graft-versus-host (GVHD) in both treatment arms in week 4. Radiance scale is shown. (**e**) Plot showing the quantification of total flux over time from live BLI of each mouse in (**d**). For panels (**d-e**) n=5 mice in each condition were used. Source data are provided in the Source Data file.

## Supplementary Figure 12

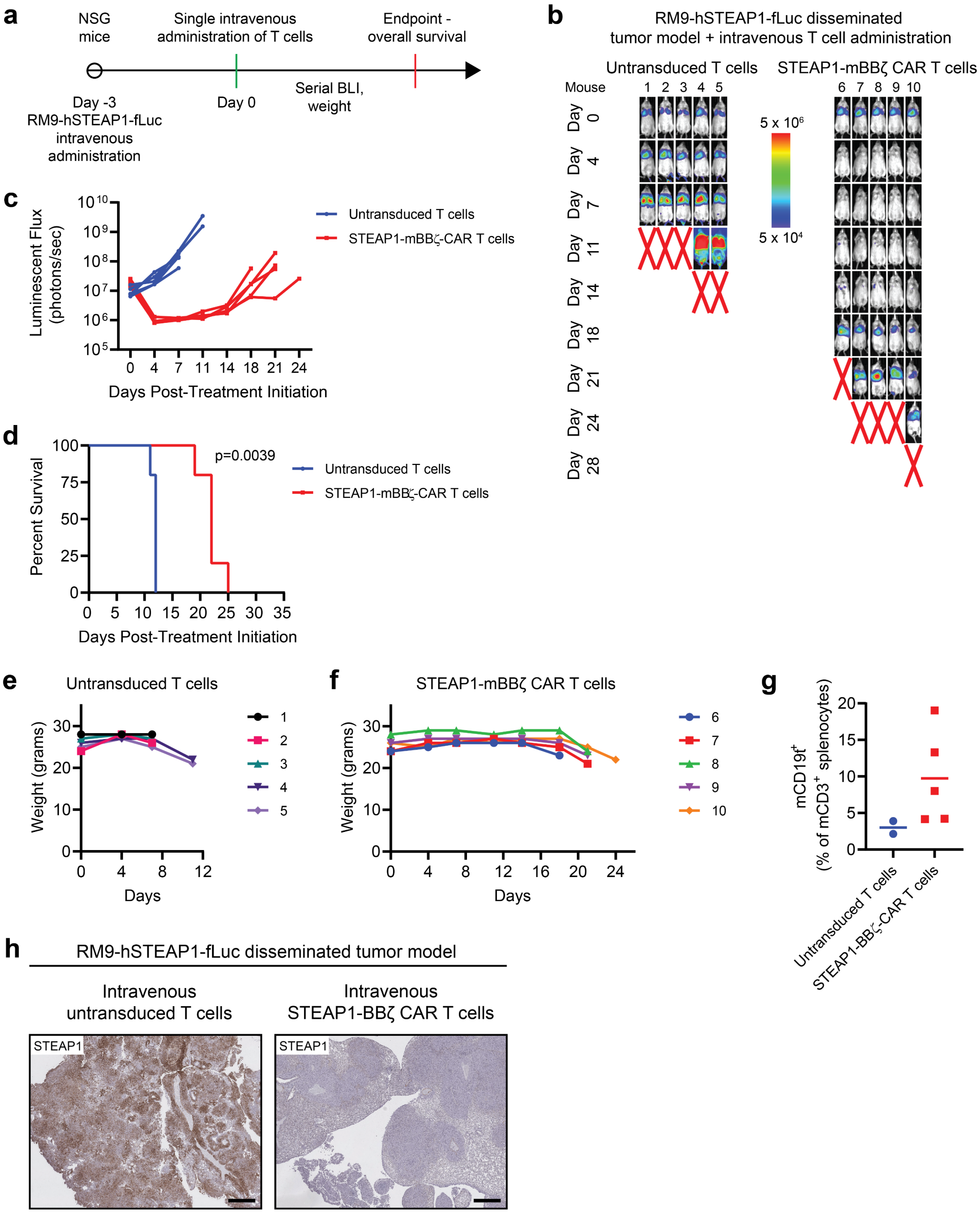


**Supplementary Figure 12. Antitumor activity of STEAP1-mBBζ CAR T cell therapy in a disseminated, non-clonal mouse RM9-hSTEAP1 prostate cancer allograft model in NSG mice.** (**a**) Schematic of tumor challenge experiments for the non-clonal RM9-hSTEAP1 disseminated model. (**b**) Serial live BLI of NSG mice engrafted with RM9-hSTEAP1-fLuc metastases and treated with a single intravenous injection of 5 x $10^6$ untransduced mouse T cells or mouse STEAP1-mBBζ CAR T cells on day 0. Red X denotes deceased mice. Radiance scale is shown. (**c**) Plot showing the quantification of total flux over time from live BLI of each mouse in (**b**). (**d**) Kaplan-Meier survival curves

of mice in (**b**) with statistical significance determined by log-rank (Mantel-Cox) test. Plots of weights for each mouse numbered in (**b**) over time in the (**e**) untransduced T cell treatment group and (**f**) STEAP1-mBBζ CAR T cell treatment group. For panels (**b-f**) n=5 mice per condition were used. (**g**) Quantification of $mCD3^{+}mCD19t^{+}$ STEAP1-mBBζ CAR T cells by flow cytometry from splenocytes of mice treated with untransduced T cells (n=2) and STEAP1-BBζ CAR T cells (n=5) collected at necropsy. (**h**) Photomicrographs at low and magnification of STEAP1 IHC staining of RM9-hSTEAP1 lung tumors after treatment with (left) mouse untransduced T cells or (right) STEAP1-BBζ CAR T cells. Scale bars = 500 µm. Immunostaining was performed on n=4 biologically independent specimens. Source data are provided in the Source Data file.

## Supplementary Figure 13

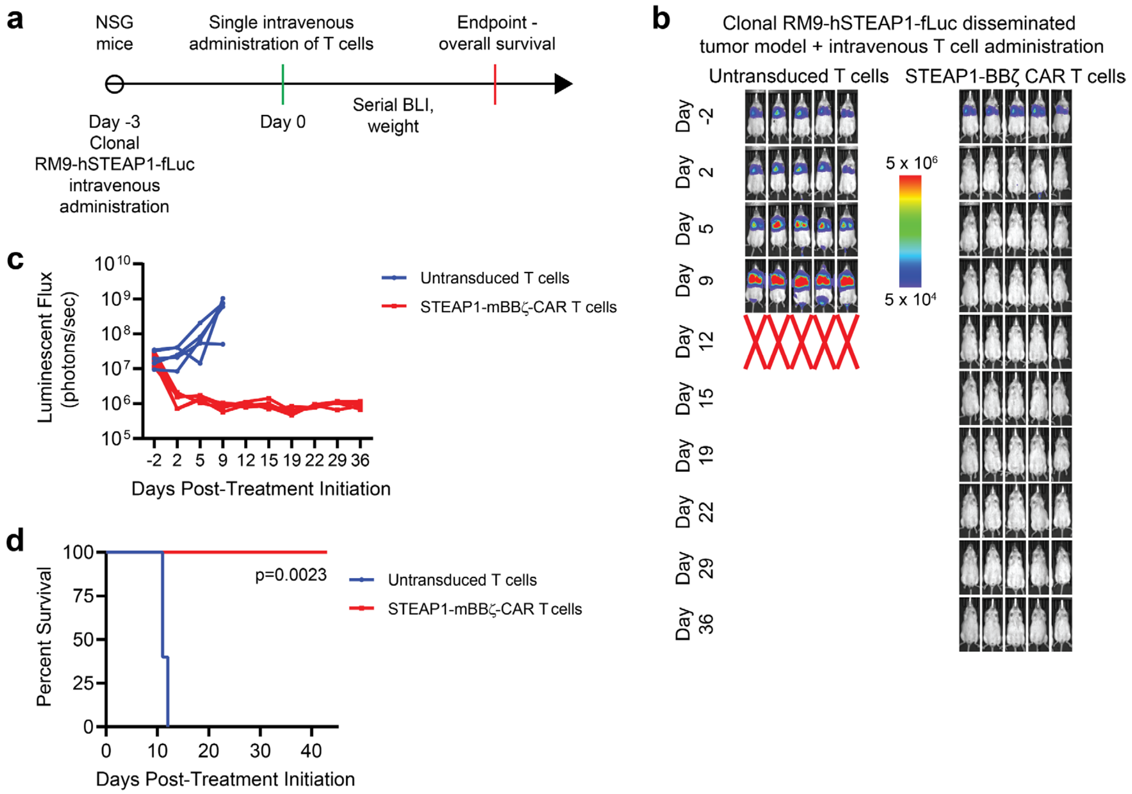


**Supplementary Figure 13. Antitumor activity of STEAP1-mBBζ CAR T cell therapy in a disseminated, clonal mouse RM9-hSTEAP1 prostate cancer allograft model in NSG mice.** (**a**) Schematic of tumor challenge experiments for the clonal RM9-hSTEAP1 disseminated model. (**b**) Serial live BLI of NSG mice engrafted with RM9-hSTEAP1-fLuc metastases and treated with a single intravenous injection of 5 x $10^6$ untransduced mouse T cells or mouse STEAP1-mBBζ CAR T cells on day 0. Red X denotes deceased mice. Radiance scale is shown. (**c**) Plot showing the quantification of total flux over time from live BLI of each mouse in (**b**). (**d**) Kaplan-Meier survival curves of mice in (**b**) with statistical significance determined by log-rank (Mantel-Cox) test. For panels (**b-d**) n=5 mice in each condition were used. Source data are provided in the Source Data file.

## Supplementary Figure 14

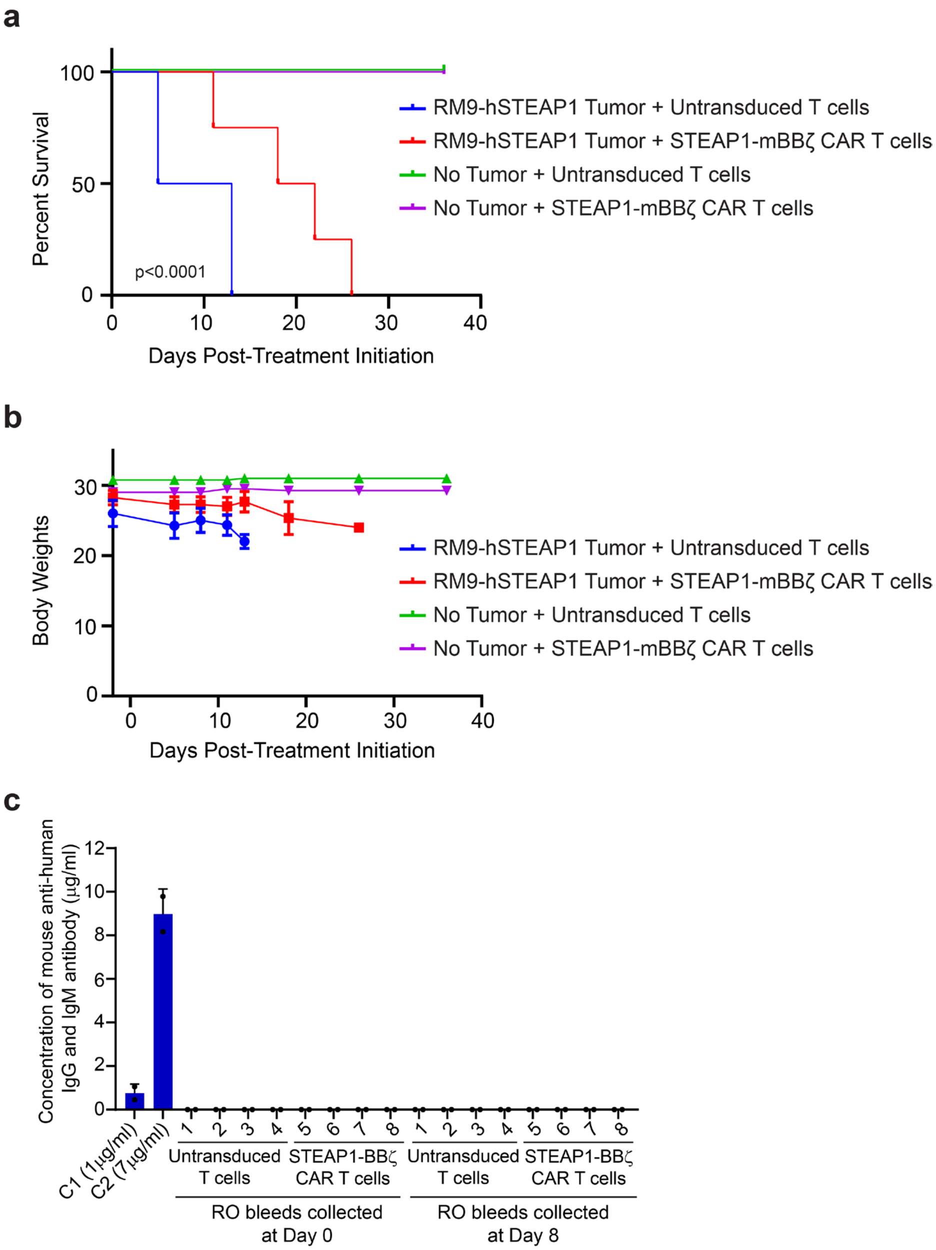


**Supplementary Figure 14. Charaterization of the safety of STEAP1-mBBζ CAR T cell therapy in non-tumor bearing hSTEAP1-KI mice.** (**a**) Kaplan-Meier survival curves of mice bearing RM9-hSTEAP1 tumors or no tumors treated with untransduced T cells or STEAP1-mBBζ CAR T cells. Statistical significance determined by log-rank (Mantel-Cox) test (p=0.0001). (**b**) Plots of average body weights of mice in each treatment group over time. n=4 mice per condition. Bars represent SD. (**c**) ELISA quantification of anti-human IgG and IgM antibodies from serum collected at day 0 and 8 from RM9-hSTEAP1 tumor bearing mice treated with untransduced T cells and STEAP1-mBBζ CAR T cells (n=4 mice per condition). Bar represents the mean with SD. Source data are provided in the Source Data file.

## Supplementary Figure 15

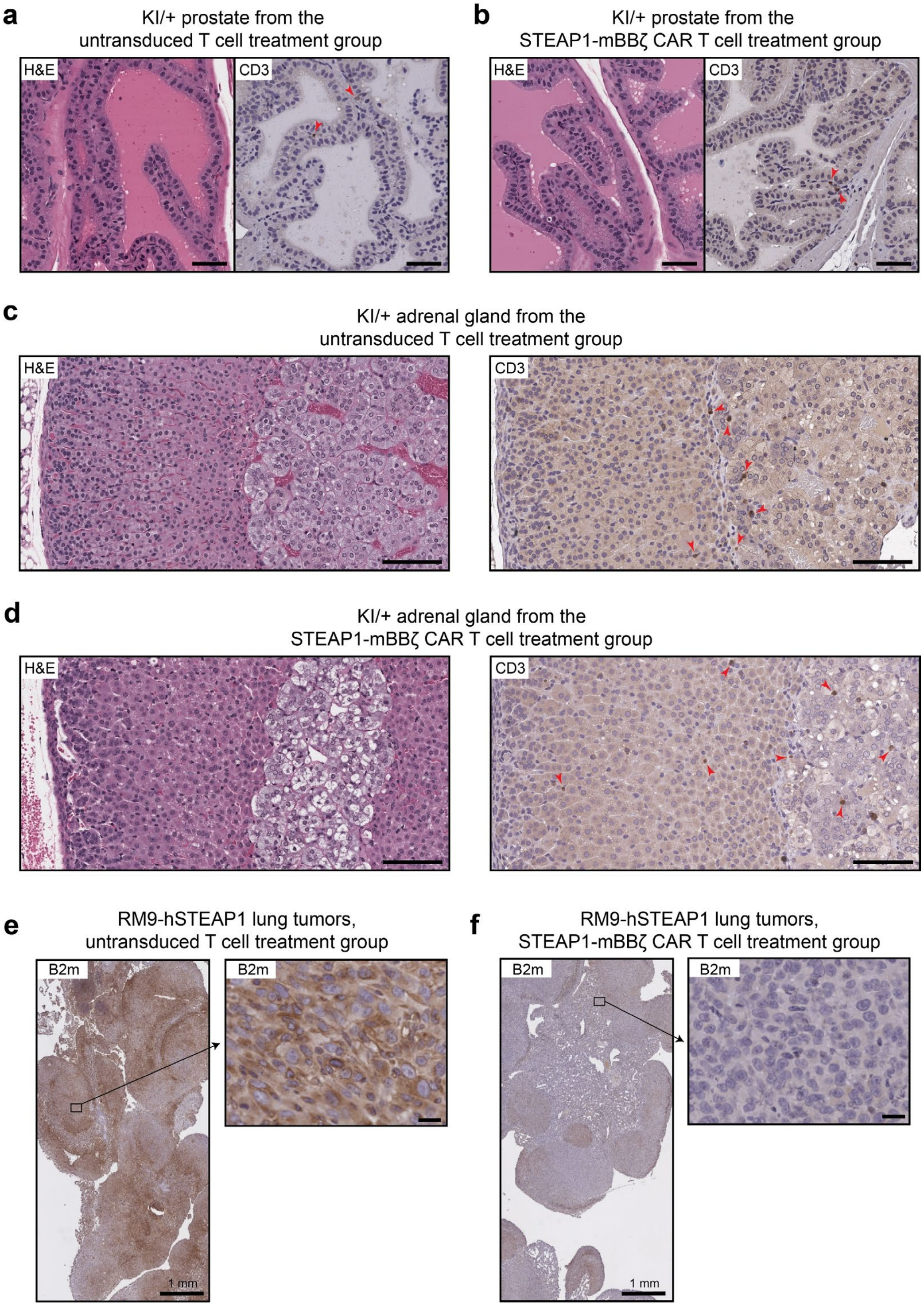


**Supplementary Figure 15. Preserved tissue architecture and absence of increased T cell infiltration in the prostates or adrenal glands of hSTEAP1-KI mice treated with mouse STEAP1-mBBζ CAR T cells.** Representative photomicrographs of hematoxylin & eosin (H&E) and CD3 IHC staining of hSTEAP1-KI/+ prostates from mice treated with (**a**) untransduced T cells and (**b**) STEAP1-mBBζ CAR T cells. Red arrowheads indicate rare $CD3^+$ cells. Scale bars = 50 μm. Representative photomicrographs of H&E and CD3 IHC staining of hSTEAP1-KI/+ adrenal glands from mice treated with (**c**) untransduced T cells and (**d**) STEAP1-mBBζ CAR T cells. Red arrowheads indicate $CD3^+$ cells.

Scale bars = 100 µm. Photomicrographs at low (left) and high (right) magnification of B2m IHC staining of RM9-hSTEAP1 lung tumors from mice after treatment with (**e**) mouse untransduced T cells and (**f**) STEAP1-mBBζ CAR T cells. Scale bars = 20 µm, unless otherwise noted. For panels (**a-f**) immunostaining was performed on n=4 biologically independent specimens.

## Supplementary Figure 16

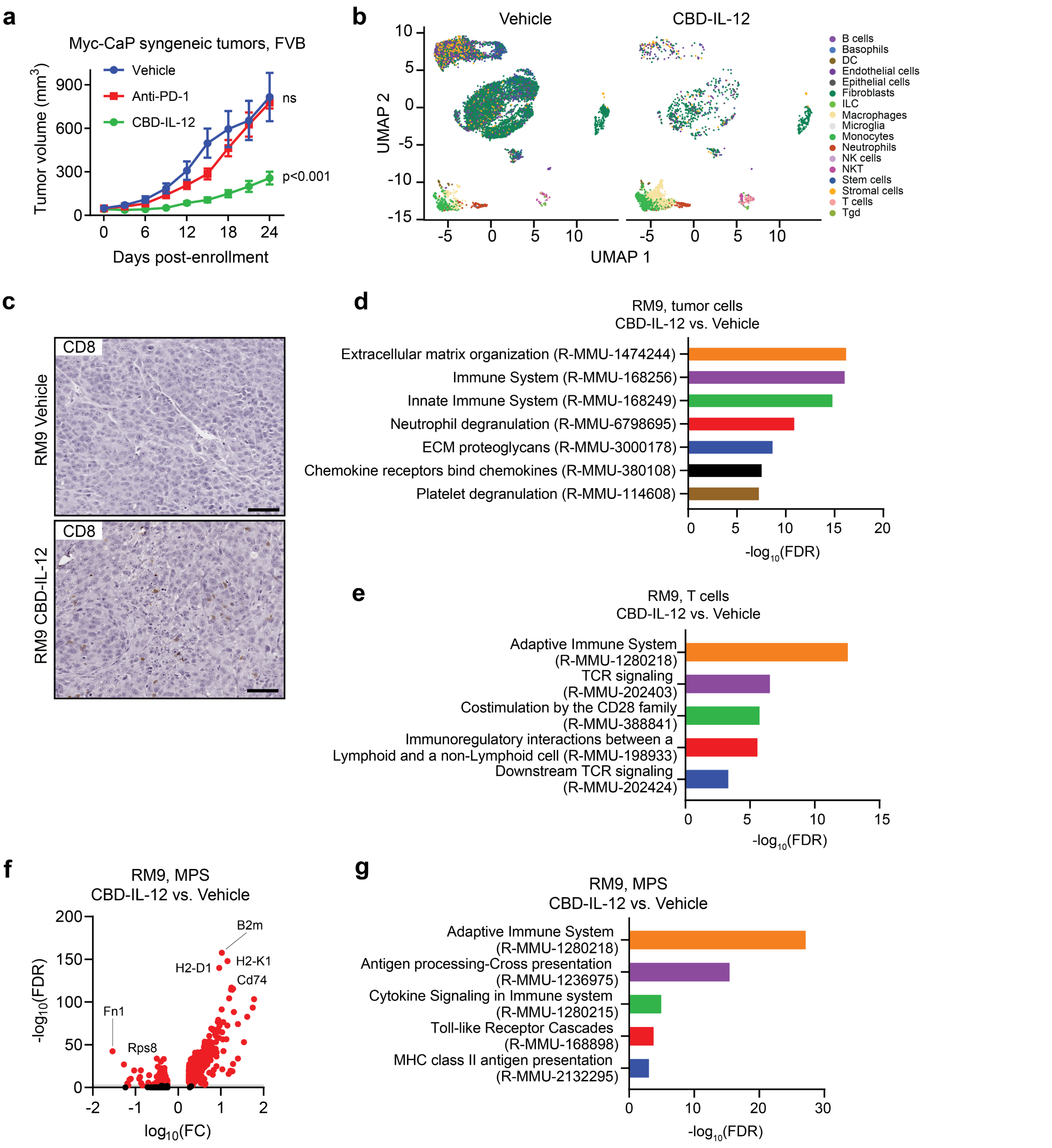


**Supplementary Fig. 16. CBD-IL-12 reprograms the tumor immune microenvironment of prostate cancer models.** (**a**) Volumes of Myc-CaP subcutaneous tumors in sygeneic FVB mice over time with treatment with vehicle (n=7 mice), anti-PD-1 (clone 29F.1A12) 200 µg (n=5 mice) by intraperitoneal injection every 5 days, or CBD-IL-12 25 µg (n=6 mice) by intravenous injection every 5 days starting on day 0. Bar represents the mean with SEM. P-values ($p<0.0001$ for vehicle vs. CDB-IL-12) were obtained from two-way ANOVA with Dunnett's multiple comparisons test. (**b**) Uniform Manifold

Approximation and Projection (UMAP) plots of single-cell RNA-seq (scRNA-seq) analysis of five RM9 tumors each aggregated from mice treated with vehicle or CBD-IL-12. (**c**) Photomicrographs of CD8 IHC staining of RM9 tumors from mice treated with (left) vehicle or CBD-IL-12 (right). Scale bars = 50 µm. CD8 immunostaining was performed on n=3 biologically independent specimens. Bar graphs showing Reactome pathways identified from differentially enriched genes ($\log_2$ fold change >1, q<0.05) expressed in (**d**) tumor cells or (**e**) T cells. FDR = false discovery rate. (**f**) Volcano plot showing differential gene expression in mononuclear phagocyte system (MPS) cells from RM9 tumors of mice treated CBD-IL-12 relative to those treated with vehicle. FC = fold change. (**g**) Bar graphs showing Reactome pathways identified from differentially enriched genes ($\log_2$ fold change >1, q<0.05) expressed in MPS cells. Source data are provided in the Source Data file.

## Supplementary Figure 17

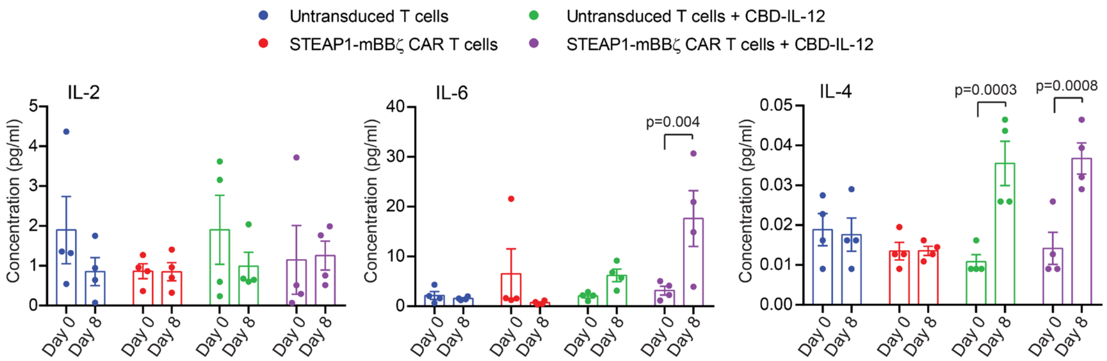


**Supplementary Fig. 17. Plasma cytokine analysis before and after mouse STEAP1-mBBζ CAR T cell therapy with or without concurrent CBD-IL-12 therapy in hSTEAP1-KI mice.** Plots showing serum cytokine levels (IL-2, IL-6, and IL-4) based on ProcartaPlex immunoassays from retroorbital bleeds of hSTEAP1-KI/+ mice bearing RM9-hSTEAP1-fLuc metastases prior to (day 0) or after (day 8) treatment with untransduced mouse T cells or mouse STEAP1-mBBζ CAR T cells with or without CBD-IL-12 treatment. n=4 for each condition. Bar represents the mean with SEM. P-values are derived from two-way ANOVA with Sidak's multiple comparisons test. Source data are provided in the Source Data file.

## Supplementary Figure 18

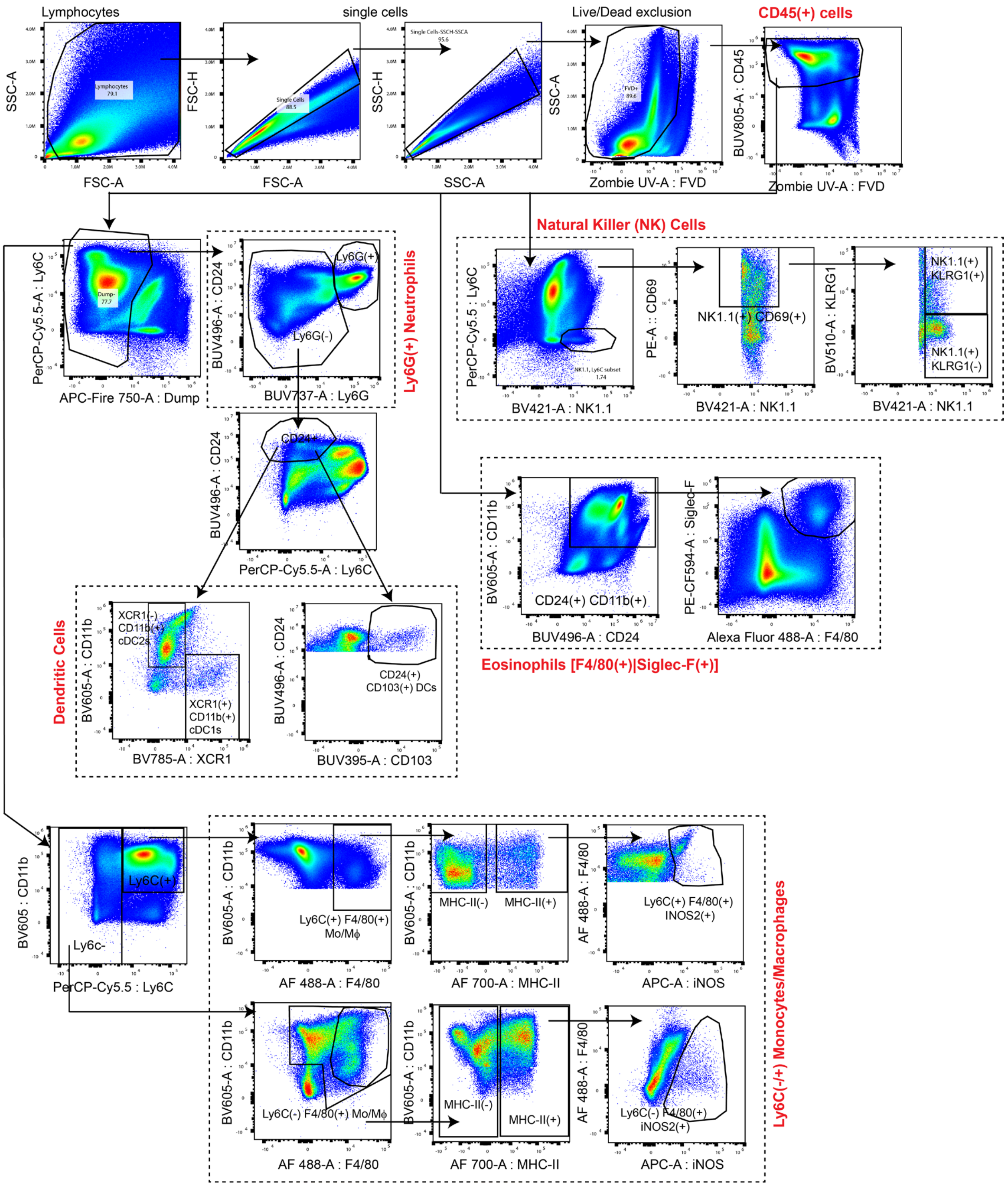


**Supplementary Figure 18. Gating strategy for the immunophenotyping of tumor immune cell subsets.** Lymphocytes were gated on forward (FSC-A) and side (SSC-A) scatter, followed by gating on single cells and live cell population based on Fixable Viability Dyes (FVD) stain. Live cells were gated for CD45$^{+}$ immune cells followed by analysis of F4/80$^{+}$Siglec-F$^{+}$ eosinophils. CD45$^{+}$ cells were selected for a dump gate based on staining for B220, CD19, CD3, CD4, and CD8a. Dump-negative cells were gated for Ly6C$^{+/-}$F4/80$^{+}$MHC-II$^{+/-}$ monocytes or macrophages, Ly6G$^{+}$ tumor associated neutrophils, CD24$^{+}$ dendritic cells, and NK1.1$^{+}$CD69$^{+}$KLRG$^{+/-}$ natural killer cells.

## Supplementary Figure 19

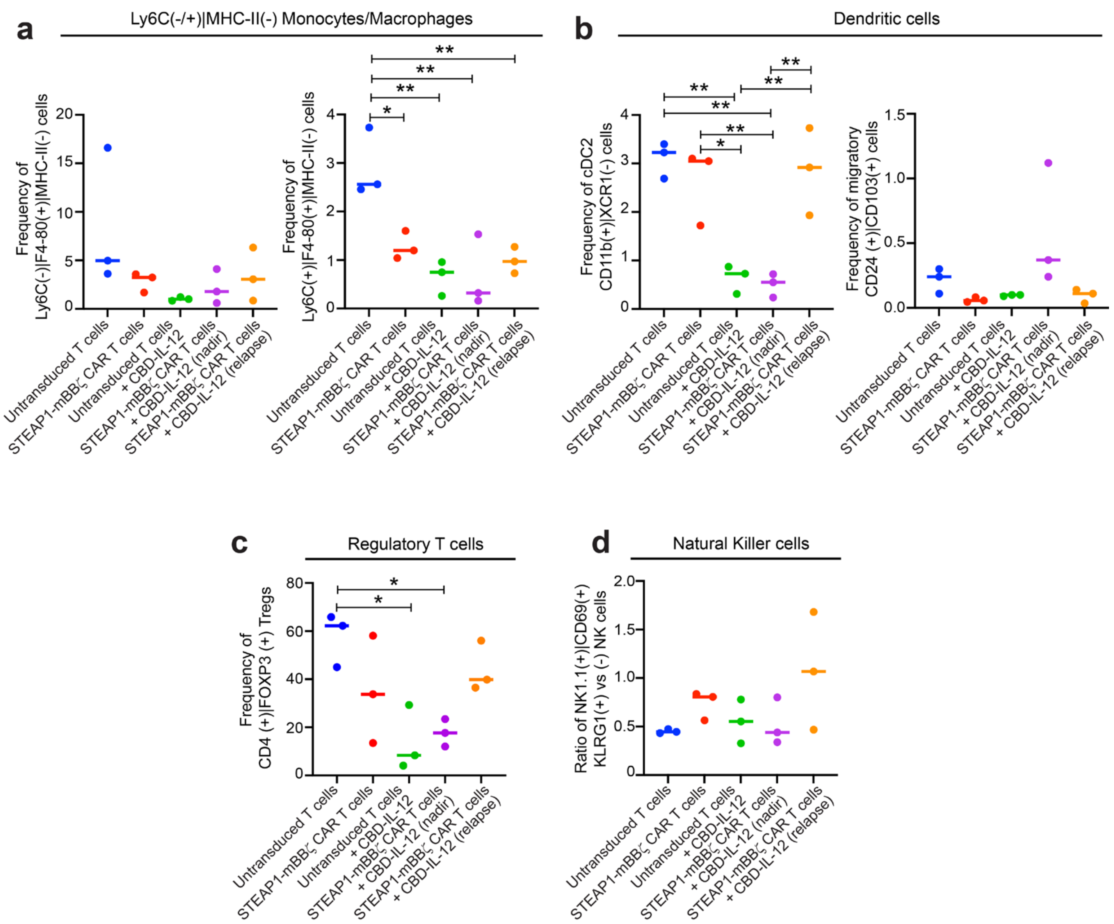


**Supplementary Figure 19. Combining CBD-IL-12 with STEAP1-mBBζ CAR T cell therapy enhances tumor infiltration of innate immune cells.** Plots showing the frequencies of (**a**) Ly6C$^-$F4/80$^+$MHC-II$^-$ (left, one-way ANOVA p=ns) and Ly6C$^+$F4/80$^+$MHC-II$^-$ (right, one-way ANOVA p=0.0017) macrophages, (**b**) CD11b$^+$XCR1$^-$ cDC2 (left, one-way ANOVA p=0.0004) and CD103$^+$ migratory dendritic cells (right, one-way ANOVA p=0.08), and (**c**) CD4$^+$FOXP3$^+$ regulatory T cells (Tregs) normalized to total CD45$^+$ cells as determined by multiparametric flow cytometry after treatment with untransduced T cells, STEAP1-mBBζ CAR T cells, untransduced T cells and CBD-IL-12, and STEAP1-mBBζ CAR T cells and CBD-IL-12 at maximal treatment response (nadir) and tumor relapse (relapse) group (one-way ANOVA p=0.015). (**d**) Plot showing the ratio of KLRG1$^+$ to KLRG1$^-$ natural killer cells (one-way ANOVA p=0.3) in the treatment groups in (**a**). For panels (**a-d**), n=3 tumors per condition, * denotes p<0.05; ** denotes p<0.01, *** denotes p<0.001. P-values are derived from one-way ANOVA with Dunn's multiple comparisons test. Source data are provided in the Source Data file.
