# Supplementary material for: Targeting advanced prostate cancer with STEAP1 chimeric antigen receptor T cell and tumor-localized IL-12 immunotherapy
Source: Nat Commun. 2023 Apr 11;14:2041. doi: 10.1038/s41467-023-37874-2 (PMC10090190; doi:10.1038/s41467-023-37874-2)
Supplement: Supplementary file 3 — Reporting Summary [file 41467_2023_37874_MOESM3_ESM.pdf]

nature portfolio

Corresponding author(s): John K. Lee
Jun Ishihara

Last updated by author(s): Mar 21, 2023

Reporting Summary

Nature Portfolio wishes to improve the reproducibility of the work that we publish. This form provides structure for consistency and transparency in reporting. For further information on Nature Portfolio policies, see our Editorial Policies and the Editorial Policy Checklist.

Statistics

For all statistical analyses, confirm that the following items are present in the figure legend, table legend, main text, or Methods section.

n/a	Confirmed	
☐	☒	The exact sample size (*n*) for each experimental group/condition, given as a discrete number and unit of measurement
☐	☒	A statement on whether measurements were taken from distinct samples or whether the same sample was measured repeatedly
☐	☒	The statistical test(s) used AND whether they are one- or two-sided<br>*Only common tests should be described solely by name; describe more complex techniques in the Methods section.*
☒	☐	A description of all covariates tested
☐	☒	A description of any assumptions or corrections, such as tests of normality and adjustment for multiple comparisons
☐	☒	A full description of the statistical parameters including central tendency (e.g. means) or other basic estimates (e.g. regression coefficient) AND variation (e.g. standard deviation) or associated estimates of uncertainty (e.g. confidence intervals)
☐	☒	For null hypothesis testing, the test statistic (e.g. *F*, *t*, *r*) with confidence intervals, effect sizes, degrees of freedom and *P* value noted<br>*Give P values as exact values whenever suitable.*
☒	☐	For Bayesian analysis, information on the choice of priors and Markov chain Monte Carlo settings
☐	☒	For hierarchical and complex designs, identification of the appropriate level for tests and full reporting of outcomes
☒	☐	Estimates of effect sizes (e.g. Cohen's *d*, Pearson's *r*), indicating how they were calculated

Our web collection on statistics for biologists contains articles on many of the points above.

Software and code

Policy information about availability of computer code

Data collection	The bulk and single-cell RNA-seq data collected for this manuscript was generated on an Illumina NovaSeq 6000 at the Genomics Shared Resource at Fred Hutchinson Cancer Center. Multiplex PCR-based mouse T cell receptor beta (TCRB) sequencing was performed using the ImmunoSeq service provided by Adaptive Biotechnologies (Seattle, USA).
Data analysis	Data and statistical analysis were performed using Microsoft Excel Office v16.65 and GraphPad Prism v9.4.1. Standard statistical tests were used to analyze biological data including Student's t test, Wilcoxon rank-sum test, Fisher's exact test, two-way ANOVA with post hoc Tukey's or Sidak's multiple comparisons. Kaplan-Meier survival curves were generated with statistical significance determined by log-rank (Mantel-Cox) test. Results for immunohistochemical (IHC) analysis were plotted or quantified using QuPath 0.2.3. Results from flow cytometric analysis were acquired using BD FACSCanto and BD FACSymphony instruments. Flow data were analyzed using FlowJo 10.8.0. Bulk and single-cell RNA-seq data were analyzed on a Linux workstation using R v4.0.3 and R studio v1.3.1093. Cytokine analyses were performed by ELISA and data were acquired using a BioTek Cytation 3 Cell Imaging Multi-Mode Microplate Reader (Agilent Technologies) or Luminex 100/200 System (Luminex). The sequencing data were processed using the following tools: Bcl2fastq2v2.20, UCSC TOIL processing pipeline v5.7.1, DESeq2 v3.15, Seurat V4, SingleR v1.10.0, and Gene Set Enrichment Analysis 4.3.1. Mouse T cell receptor beta sequencing data was analyzed using immunoSEQ Analyzer 3.0 (Adaptive Biotechnologies). For functional experiments, each was repeated at least three times independently and results were expressed as mean ± SD or mean ± SEM.

For manuscripts utilizing custom algorithms or software that are central to the research but not yet described in published literature, software must be made available to editors and reviewers. We strongly encourage code deposition in a community repository (e.g. GitHub). See the Nature Portfolio guidelines for submitting code & software for further information.

Data

Policy information about availability of data

All manuscripts must include a data availability statement. This statement should provide the following information, where applicable:

- Accession codes, unique identifiers, or web links for publicly available datasets
- A description of any restrictions on data availability
- For clinical datasets or third party data, please ensure that the statement adheres to our policy

The bulk and single cell RNA-Seq data from this study has been submitted to the NCBI Gene Expression Omnibus (GEO) under the accession number (GSE214585).
The link to the following are:
Superseries:
https://www.ncbi.nlm.nih.gov/geo/query/acc.cgi?&acc=GSE214585
Subseries:
For RNA-Seq
https://www.ncbi.nlm.nih.gov/geo/query/acc.cgi?acc=GSE214583
For scRNA-Seq
https://www.ncbi.nlm.nih.gov/geo/query/acc.cgi?acc=GSE214584
For TCR beta sequencing
https://www.ncbi.nlm.nih.gov/geo/query/acc.cgi?acc=GSE228884

Human research participants

Policy information about studies involving human research participants and Sex and Gender in Research.

Reporting on sex and gender	NA
Population characteristics	NA
Recruitment	NA
Ethics oversight	NA

Note that full information on the approval of the study protocol must also be provided in the manuscript.

Field-specific reporting

Please select the one below that is the best fit for your research. If you are not sure, read the appropriate sections before making your selection.

☒ Life sciences ☐ Behavioural & social sciences ☐ Ecological, evolutionary & environmental sciences

For a reference copy of the document with all sections, see nature.com/documents/nr-reporting-summary-flat.pdf

Life sciences study design

All studies must disclose on these points even when the disclosure is negative.

Sample size	In vitro experiments were performed using three technical replicates and each experiment was repeated at least three times. Sample sizes for in vitro experiments were chosen based on the standard in the field. For in vivo studies, samples size were anticipated for an effect size of >0.8 with an alpha error probability of 0.05 at a power of 80%. Thus the recommended sample size in all the mice experiments performed were kept to at least 4-5 animals per group.
Data exclusions	No data was excluded from the analyses.
Replication	Experiments have been repeated multiple times using different independent biological samples with similar experimental conditions or otherwise mentioned in the figure legends, main text or methods.
Randomization	For mice xenograft and allograft experiments, tumor bearing mice were randomized into groups before treatment.
Blinding	Blinding and subsequent decoding of specimens were performed for immunohistochemical scoring of prostate cancer patients' tissue microarrays and mouse xenograft and allograft tumors. Also, for quantification experiments, such as scoring immunohistochemistry, experimental conditions were blinded during analysis.

Reporting for specific materials, systems and methods

We require information from authors about some types of materials, experimental systems and methods used in many studies. Here, indicate whether each material, system or method listed is relevant to your study. If you are not sure if a list item applies to your research, read the appropriate section before selecting a response.

Materials & experimental systems

n/a	Involved in the study	
☐	☒	Antibodies
☐	☒	Eukaryotic cell lines
☒	☐	Palaeontology and archaeology
☐	☒	Animals and other organisms
☒	☐	Clinical data
☒	☐	Dual use research of concern

Methods

n/a	Involved in the study	
☒	☐	ChIP-seq
☐	☒	Flow cytometry
☒	☐	MRI-based neuroimaging

Antibodies

Antibodies used

All the antibodies used in the study are listed below:
1. Human CD3 conjugated to APC-eFluor780 (Clone SK7, Thermo Fisher, 47-0036-42)
2. Mouse anti-human CD8 conjugated to FITC (Clone RPA-T8, BD Biosciences, 555366)
3. Rabbit anti-human EGFR (cetuximab) conjugated to PE (Clone C225, Novus Biologicals, NBP2-52671PE)
4. Mouse anti-human CD3 conjugated to BUV395 (Clone UCHT1, BD Biosciences, 563548)
5. Mouse anti-human CD4 conjugated to BV605 (Clone SK3, BioLegend, 344645)
6. Mouse anti-human CD8 conjugated to BUV805 (Clone SK1, BD Biosciences, 564912)
7. Mouse anti-human CD45RO conjugated to BV510 (Clone UCHL1, BioLegend, 304246)
8. Mouse anti-human CD45RA conjugated to BV711 (Clone HI100, BioLegend, 304138)
9. Mouse anti-human PD-1 conjugated to APC (Clone A17188B, BioLegend, 621610)
10. Mouse anti-human CD95 conjugated to BUV615 (Clone DX2 (RUO), BD Biosciences, 752346)
11. Mouse anti-human CXCR3 conjugated to BV421 (Clone G025H7, BioLegend, 353716)
12. Mouse anti-human CD62L conjugated to BV785 (Clone SK11, BD Biosciences, 565311)
13. Mouse anti-human LAG-3 conjugated to BV421 (Clone T47-530 (RUO), BD Biosciences, 565721)
14. Mouse CD8a conjugated to FITC (Clone 53-6.7, BioLegend, 100706)
15. Rat anti-mouse CD19 conjugated to PE (Clone 6D5, BioLegend, 115508)
16. Rat anti-human IgG Fc antibody conjugated to APC (BioLegend, 410712)
17. Mouse anti-STEAP (B-4) (Santa Cruz, sc-271872, 1:1,000)
18. GAPDH (Clone GT239, GeneTex, GX627408, 1:5,000)
19. Goat anti-mouse IgG (H+L) secondary antibody conjugated with horseradish peroxidase (Thermo Fisher, 31430, 1:10,000)
20. Rabbit anti-STEAP1 IgG polyclonal antibody (LS Bio, LS-C291740, 1:500)
21. Rabbit anti-CD3 antibody (Clone SP7, Thermo Fisher, MA5-14524, 1:100)
22. Mouse anti-PSMA antibody (Clone 3E6, Dako, M3620, 1:50)
23. Rabbit anti-beta-2-microglobulin antibody (Clone EPR21752-214, Abcam, ab218230, 1:1000)
24. Mouse anti-HLA Class 1 ABC antibody (Clone EMR8-5, Abcam, ab70328, 1:3000)
25. Hamster Anti-Mouse CD103 conjugated to BUV395 (Clone 2E7, BD Biosciences, 748253)
26. Anti-Mouse FOXP3 Monoclonal Antibody (Clone FJK-16s), eFluor™ 450, eBioscience
27. CD24 Rat anti-Mouse, BUV496, Clone: M1/69, BD Horizon, 612953
28. BUV737 Rat Anti-Mouse Ly-6G Clone 1A8 (RUO), (741813, BD Biosciences)
29. BUV805 Rat Anti-Mouse CD45 Clone 30-F11 (RUO), (748370, BD Biosciences)
30. BV 421 anti-mouse NK-1.1 Clone PK136, (108731, BioLegend)
31. BV510 Hamster Anti-Mouse KLRG1 Clone 2F1 (RUO), (740156, BD Biosciences)
32. Brilliant Violet 605™ anti-mouse/human CD11b Clone M1/70, (101237, BioLegend)
33. BV650 Mouse Anti-Ki-67 Clone B56 (RUO), (563757, BD Biosciences)
34. Brilliant Violet 785™ anti-mouse/rat XCR1, (148225, BioLegend)
35. Alexa Fluor™ 488 Rat Anti-Mouse F4/80 Clone T45-2342 (RUO)(567201, BD Biosciences)
36. PerCP/Cyanine5.5 anti-mouse Ly-6C Antibody Clone HK1.4 (128011, BioLegend)
37. PE anti-mouse CD69 Clone H1.2F3 (104507, BioLegend)
38. PE-CF594 Rat Anti-Mouse Siglec-F Clone E50-2440 (RUO)(562757, BD Biosciences)
39. iNOS Monoclonal Antibody (Clone CXNFT), APC, (17-5920-82, eBioscience)
40. Alexa Fluor® 700 anti-mouse I-A/I-E (MHC class II) Clone M5/114.15.2 (107622, BioLegend)
41. APC/Fire™ 750 anti-mouse CD4 Clone RM4-4 (116019, BioLegend)
42. APC/Fire™ 750 anti-mouse CD8a Clone 53-6.7 (100765, BioLegend)
43. Rabbit monoclonal Anti-Androgen Receptor antibody (Clone EPR1535(2), Abcam (ab133273), 1:1000)
44. Mouse anti-SYP/Synaptophysin Antibody (Santa Cruz, sc-17750 (D4), 1:50)
45. Polyclonal Rabbit anti-STEAP1 antibody (BioRad, AHP1438)

46. CD45R (B220) Monoclonal Antibody (RA3-6B2), APC-eFluor™ 780, (47-0452-82, eBioscience)
47. APC/Fire™ 750 anti-mouse CD19 Antibody (Clone 6D5), (115558, BioLegend)
48. APC/Fire™ 750 anti-mouse CD3 Antibody (Clone 145-2C11), (100362, BioLegend)

Validation

For each antibody, the validation statement has been taken from the manufacturer's website or data sheet and detailed as follows:
1. Human CD3 conjugated to APC (Thermo Fisher, 47-0036-42): Validated for staining normal human peripheral blood cells with Anti-Human CD3 APC-eFluor® 780 by FACS.
https://www.thermofisher.com/antibody/product/CD3-Antibody-clone-SK7-Monoclonal/47-0036-42
2. Mouse anti-human CD8 conjugated to FITC (BD Biosciences, 555366):
This clone has been reported to react with a subset of peripheral blood lymphocytes, but not monocytes nor granuloyctes, of baboon and both rhesus and cynomolgus macaque monkey by FACS.
https://www.bdbiosciences.com/en-eu/products/reagents/flow-cytometry-reagents/research-reagents/single-color-antibodies-ruo/fitc-mouse-anti-human-cd8.555366
3. Rabbit anti-human EGFR (cetuximab) conjugated to PE (Novus Biologicals, NBP2-52671PE)
Not validated.
4. Mouse anti-human CD3 conjugated to BUV395 (BD Biosciences, 563548): Validated by staining Human whole blood by FACS.
https://www.bdbiosciences.com/en-us/products/reagents/flow-cytometry-reagents/research-reagents/single-color-antibodies-ruo/buv395-mouse-anti-human-cd3.563546?gclid=CjwKCAjwhNWZBhB_EiwAPzlhNteOv9W616Yvv-O7JC1st4uW0K4MR7eG8R_KdwauxJSWGkAIQ1btsRoCT1cQAvD_BwE
5. Mouse anti-human CD4 conjugated to BV605 (BioLegend, 344645): Validated by staining Human peripheral blood by FACS.
https://www.biolegend.com/ja-jp/products/brilliant-violet-605-anti-human-cd4-antibody-15992
6. Mouse anti-human CD8 conjugated to BUV805 (BD Biosciences, 564912): Validated by staining Human peripheral blood by FACS.
https://www.bdbiosciences.com/en-us/products/reagents/flow-cytometry-reagents/research-reagents/single-color-antibodies-ruo/buv805-mouse-anti-human-cd8.612890?gclid=CjwKCAjwhNWZBhB_EiwAPzlhNlT011vVhliIOHFZcyPhvXHw30vrpZ2_eQKW7zuYQkbh2PVhExX85BoClY8QAvD_BwE
7. Mouse anti-human CD45RO conjugated to BV510 (BioLegend, 304246): Validated by staining Human peripheral blood lymphocytes stained with CD45RA PE and CD45RO (clone UCHL1) by FACS.
https://www.biolegend.com/fr-ch/products/brilliant-violet-510-anti-human-cd45ro-antibody-11922
8. Mouse anti-human CD45RA conjugated to BV711 (BioLegend, 304138): Validated by staining Human peripheral blood lymphocytes stained with CD45RA (clone HI100) Brilliant Violet 711 by FACS.
https://www.biolegend.com/ja-jp/products/brilliant-violet-711-anti-human-cd45ra-antibody-7937
9. Mouse anti-human PD-1 conjugated to BV421 (BD Biosciences, 565935): Validated by Human whole blood staining with the BD HorizonTM BV421 Mouse Anti-Human CD279 (PD-1) antibody by FACS.
https://www.bdbiosciences.com/content/dam/bdb/products/global/reagents/flow-cytometry-reagents/research-reagents/single-color-antibodies-ruo/562516_base/pdf/562516.pdf
10. Mouse anti-human CD95 conjugated to BUV615 (BD Biosciences, 752346): The antibody has been validated by FACS in four publications mentioned on the manufacturer's website.
https://www.bdbiosciences.com/en-us/products/reagents/flow-cytometry-reagents/research-reagents/single-color-antibodies-ruo/buv615-mouse-anti-human-cd95.752346
11. Mouse anti-human CXCR3 conjugated to BV421 (BioLegend, 353716): Validated by staining Human peripheral blood lymphocytes with CXCR3 (clone G025H7) Brilliant Violet 421™ by FACS.
https://www.biolegend.com/nl-be/products/brilliant-violet-421-anti-human-cd183-cxcr3-antibody-7712
12. Mouse anti-human CD62L conjugated to BV785 (BD Biosciences, 565311): Validated by staining human peripheral blood lymphocytes with the BD Horizon™ BV786 Mouse Anti-Human CD127 antibody by FACS.
https://www.bdbiosciences.com/en-us/products/reagents/flow-cytometry-reagents/research-reagents/single-color-antibodies-ruo/bv786-mouse-anti-human-cd127.563324?gclid=CjwKCAjwhNWZBhB_EiwAPzlhNhc1h-v1GsAeIEU40Rl8l6-bIK-OL9465KbRcXrTdO_mcqagivkOqhoCDvEQAvD_BwE
13. Mouse anti-human LAG-3 conjugated to BV421 (BD Biosciences, 565721): Validated by multicolor flow cytometric analysis of LAG-3 expression on unstimulated and stimulated human peripheral blood lymphocytes. Human peripheral blood mononuclear cells (PBMC) were cultured for 3 days with plate-bound Anti-Human CD3 (10 µg/mL for coating) and soluble Anti-Human CD28 (Cat. No. 555725; 1 µg/mL) antibodies, and Human Recombinant IL-2.
https://www.bdbiosciences.com/en-ca/products/reagents/flow-cytometry-reagents/research-reagents/single-color-antibodies-ruo/bv421-mouse-anti-human-lag-3-cd223.565721
14. Mouse CD8a conjugated to FITC (BioLegend, 100706): Validated by staining C57BL/6 mouse splenocytes with CD8 (clone 53-6.7) FITC by FACS.
https://www.biolegend.com/fr-fr/products/fitc-anti-mouse-cd8a-antibody-153
15. Rat anti-mouse CD19 conjugated to PE (BioLegend, 115508): Validated by staining C57BL/6 mouse splenocytes with CD19 (clone 6D5) PE by FACS.
https://www.biolegend.com/fr-lu/products/pe-anti-mouse-cd19-antibody-1530
16. Rat anti-human IgG Fc antibody conjugated to APC (BioLegend): Validated by staining Human peripheral blood lymphocytes were stained with CD19 FITC and IgG (clone M1310G05) APC by FACS.
https://www.biolegend.com/en-ie/products/apc-anti-human-igg-fc-11935
17. Mouse anti-STEAP (Santa Cruz, sc-271872, 1:1,000): Validated by Western blot analysis of STEAP expression in LNCaP whole cell lysate.
https://www.scbt.com/p/steap-antibody-b-4
18. GAPDH (GeneTex, GX627408, 1:5,000): Validated by western blotting, Immunohistochemistry (ICC) and immunofluorescence (IF).
https://www.genetex.com/Product/Detail/GAPDH-antibody-GT239/GTX627408#datasheet
19. Goat anti-mouse IgG (H+L) secondary antibody conjugated with horseradish peroxidase (Thermo Fisher, 31430, 1:10,000): Validated by western blotting, ICC, IHC and ELISA.
https://www.thermofisher.com/antibody/product/Goat-anti-Mouse-IgG-H-L-Secondary-Antibody-Polyclonal/31430
20. Rabbit anti-STEAP1 antibody (LS Bio, LS-C291740, 1:500): Validated for Peptide-ELISA, IHC and WB.

https://www.lsbio.com/antibodies/steap1-antibody-steap-antibody-aa31-80-ihc-wb-western-ls-c291740/301281
21. Rabbit anti-CD3 antibody (Thermo Fisher, MA5-14524, 1:100): Validated for WB, IHC and FACS.
https://www.thermofisher.com/antibody/product/CD3e-Antibody-clone-SP7-Monoclonal/MA5-14524
22. Mouse anti-PSMA antibody (Dako, M3620, 1:50): Validated for WB and IHC.
https://www.agilent.com/en/product/immunohistochemistry/antibodies-controls/primary-antibodies/prostate-specific-membrane-antigen-%28concentrate%29-76591
23. Rabbit anti-beta-2-microglobulin antibody (Abcam, ab218230): Validated for WB, IHC-P, IP, FACS in B2M knockout cells.
https://www.abcam.com/beta-2-microglobulin-antibody-epr21752-214-ab218230.html
24. Mouse anti-HLA Class 1 ABC antibody (Abcam, ab70328): Validated for WB, IHC, FACS.
https://www.abcam.com/hla-class-1-abc-antibody-emr8-5-ab70328.html
(25-42) and (46-48). Antibodies used for mouse TME immuno-phenotyping were tested on mouse splenocytes and with Fluorescence Minus One (FMO) controls for each antibodies used in the panel was standardized in-house. Detailed references are available at the website on the indicated catalog numbers.
43. Rabbit Monoclonal Anti-Androgen Receptor antibody (ab133273): Validated for WB, IHC-P, ICC/IF
https://www.abcam.com/products/primary-antibodies/androgen-receptor-antibody-epr15352-ab133273.html
44. Mouse anti-SYP/Synaptophysin Antibody (Santa Cruz, sc-17750 (D4))
SYP/Synaptophysin Antibody (D-4) is recommended for detection of SYP of mouse, rat and human origin by WB, IP, IF, IHC(P) and ELISA, https://www.scbt.com/p/syp-antibody-d-4?gclid=CjwKCAjwq-WgBhBMEiwAzKSH6EtbFipEUtoFanIV1-nCWx82aAZo2LvcIbUcx_rqmOv0OEXkol0PjhoClE0QAvD_BwE
45. Polyclonal Rabbit anti-STEAP1 antibody (BioRad, AHP1438) - Antibody validated by WB and IHC.
https://www.bio-rad-antibodies.com/polyclonal/human-steap1-antibody-ahp1438.html?f=purified&JSESSIONID_STERLING=9D13D4A574B54BDE7CD387D95E4C0F19.ecommerce1&evCntryLang=US-en&cntry=US&thirdPartyCookieEnabled=true

Eukaryotic cell lines

Policy information about cell lines and Sex and Gender in Research

Cell line source(s)	22Rv1 (CRL-2505), LNCaP (CRL-1740), PC3 (CRL-1435), DU145 (HTB-81), NCI-H660 (CRL-5813), C4-2B (CRL-3315), RM9 (RL-3312), HEK293T (CRL-3216), and Myc-CaP (CRL-3255) were obtained from the American Type Culture Collection. LNCaP95 cells were a gift from Stephen R. Plymate (University of Washington, Seattle). MSKCC EF1 were derived from the MSKCC PCa4 organoid line provided by Yu Chen (Memorial Sloan Kettering Cancer Center). Cell lines were maintained in RPMI 1640 or DMEM medium supplemented with 10% FBS, 100 U/mL penicillin and 100 µg/mL streptomycin, and 4 mmol/L GlutaMAX (Thermo Fisher). PLAT-E (RV-101) Retroviral packaging cell line was obtained from Cell Biolabs, Inc. and were cultured in DMEM+10% FBS media.
Authentication	Cell line authentication was done via short tandem repeat (STR) profiling at the IDEXX BioAnalytics, 4011 Discovery Drive, Columbia, MO 65201
Mycoplasma contamination	All cell lines routinely tested negative for Mycoplasma contamination.
Commonly misidentified lines (See ICLAC register)	No misidentified cell lines were used in this study.

Animals and other research organisms

Policy information about studies involving animals; ARRIVE guidelines recommended for reporting animal research, and Sex and Gender in Research

Laboratory animals	For studies using immunocompromised mice, six- to eight-week-old male NSG (NOD-SCID-IL2Rγ-null) mice were obtained from The Jackson Laboratory and were 2-4 months old when used for the studies.<br>Six- to eight-week-old wild-type C57Bl/6 (C57Bl/6J, Strain #000664) and FVB (FVB/NJ, Strain #001800) mice were obtained from The Jackson Laboratory and were 2-4 months old when used for the studies.<br>For animal studies using hSTEAP1-KI mice, heterozygous hSTEAP1-KI mice were generated by crossing homozygous hSTEAP1-KI mice with wild-type C57Bl/6 mice. For mice experiments with hSTEAP1-KI mice, mice 2-4 months old were used for the studies.
Wild animals	The study did not involve wild animals.
Reporting on sex	As prostate cancer is a disease afflicting men only male mice were used for all the experiments. Female STEAP1 knock-in mice were used for the purpose of breeding.
Field-collected samples	The study did not include field-collected samples.
Ethics oversight	All mouse studies were performed in accordance with protocols approved by the Fred Hutchinson Cancer Center Institutional Animal Care and Use Committee and regulations of Comparative Medicine.

Note that full information on the approval of the study protocol must also be provided in the manuscript.

Flow Cytometry

Plots

Confirm that:

- [x] The axis labels state the marker and fluorochrome used (e.g. CD4-FITC).
- [x] The axis scales are clearly visible. Include numbers along axes only for bottom left plot of group (a 'group' is an analysis of identical markers).
- [x] All plots are contour plots with outliers or pseudocolor plots.
- [x] A numerical value for number of cells or percentage (with statistics) is provided.

Methodology

Sample preparation	For immunophenotype analysis, T cells were counted and the required number of cells for each experiment (as described in detailed methodology) was washed with cold PBS, followed by staining with specific antibodies of interest. Cells were incubated on ice for 30 minutes for antibody staining. After incubation, stained cells were washed twice with cold PBS to wash off non-specific antibody binders. Samples were kept on ice until analysis and analyzed within an hour of staining.
Instrument	Results from flow cytometric analysis were acquired using BD FACSCanto and BD FACSymphony instruments. A Sony MA900 Multi-Application Cell Sorter was used for any experiments involving the sorting of cells.
Software	FlowJo 10.8.0 software.
Cell population abundance	Flow cytometry experiments were performed either on pure populations of isogenic cell lines or on T cells isolated from the peripheral blood mononuclear cells (PBMCs) of different human donors. Purity was determined to be >95% in all the analyzed samples.

Gating strategy

Immunophenotyping CAR T cell products and assessment of peripheral persistence:

Setting up compensation controls: Appropriate compensation controls for each fluorochromes (FITC, APC-Cy7, PE) were generated using compensation beads. Unstained control and individual fluorochrome conjugated antibody-stained beads were used to calibrate and set voltages.

Flow cytometric analysis for immunophenotyping T cells was performed by staining 200,000 cells for each condition to be evaluated. The details of staining are mentioned in the Material and Methods section. After setting up the compensation, the following strategy was used to analyze the test samples. Preliminary FSC-A vs. SSC-A plots were used to gate on the cell population. SSC-H vs. SSC-A (or FSC-H vs. FSC-H) plots were used to gate the single cell population (or to eliminate the doublets). FITC (CD8) vs. APC-Cy7 (CD3) and PE (transduction marker: EGFR-t for human T cells; CD19-t for murine T cells) vs. APC-Cy7 (CD3) plots were used to set up gates for determining the percentages of transduced/untransduced T cells. All the samples were recorded on the same settings. FCS files were exported and analyzed using FlowJo v10.8.0.

Gating strategy for analysis of stem-like T cell populations and assessment of exhaustion markers: A multiplex flow panel was developed for this analysis with details of staining and antibodies provided in the Material and Methods section. The gating strategy for the analysis of stem-like T cells was adapted from Rosenberg et al. Adoptive cell transfer as personalized immunotherapy for human cancer. Science. 2015 Apr 3;348(6230):62-8. Briefly, after setting up the voltages based on compensation, preliminary FSC-A vs. SSC-A plots were used to gate on the cell population. SSC-H vs. SSC-A (or FSC-H vs. FSC-H) plots were used to gate the single cell population (or to eliminate the doublets). Viability stain vs. CD3 plots were used to gate CD3+ T cells. Next, the CD4+ and CD8+ T cells populations were gated for the T cell transduction marker (EGFRt-PE+ and EGFRt-PE-) populations. Both of these populations were gated for CCR7 vs. CD45RO to select CCR7+/CD45RO- T naïve and stem-like populations. This population was further gated on CD45RA vs. CD62L and CD95 vs. CXCR3 to obtain stem-like T cell (CD45RA+CD62L+CD95+CXCR3+) populations.

For the evaluation of T cell exhaustion markers, cell surface staining for PD1, LAG3 along with CD3, CD4, CD8 and EGFRt was performed as described in the Material and Methods section. CD4+/EGFRt+ and CD8+/EGFRt+ cells of interest were gated as described above. SSC-A vs. PD1 and SSC-A vs. LAG3 plots were used to gate the PD1+ and LAG3+ populations. PHA-L stimulated and unstimulated cells were used as positive and negative controls for this experiment.

A detailed gating strategy for the analysis of tumor immune cell subsets is provided as Supplementary Fig. 18.

- [x] Tick this box to confirm that a figure exemplifying the gating strategy is provided in the Supplementary Information.
